# Development of prognostic signatures for intermediate-risk papillary thyroid cancer

**DOI:** 10.1186/s12885-016-2771-6

**Published:** 2016-09-15

**Authors:** Kevin Brennan, Christopher Holsinger, Chrysoula Dosiou, John B. Sunwoo, Haruko Akatsu, Robert Haile, Olivier Gevaert

**Affiliations:** 1 Division of Biomedical Informatics Research, Department of Medicine, Stanford University, 1265 Welch Road, Stanford, CA 94305-5479 USA; 2 Division of Head and Neck Surgery, Department of Otolaryngology—Head and Neck Surgery, Stanford University, 801 Welch Road, Stanford, CA 94304-1611 USA; 3 Division of Endocrinology, Department of Medicine, Stanford University, 300 Pasteur Drive, Stanford, CA 94305-5103 USA; 4 Division of Oncology, Department of Medicine, Stanford University, 265 Campus Drive, Stanford, CA 94305-5458 USA; 5 Stanford Center for Biomedical Informatics Research, Department of Medicine & Department of Biomedical Data Science, Stanford University, Stanford, USA

**Keywords:** Papillary thyroid cancer, Prognosis, Gene expression

## Abstract

**Background:**

The incidence of Papillary thyroid carcinoma (PTC), the most common type of thyroid malignancy, has risen rapidly worldwide. PTC usually has an excellent prognosis. However, the rising incidence of PTC, due at least partially to widespread use of neck imaging studies with increased detection of small cancers, has created a clinical issue of overdiagnosis, and consequential overtreatment. We investigated how molecular data can be used to develop a prognostics signature for PTC.

**Methods:**

The Cancer Genome Atlas (TCGA) recently reported on the genomic landscape of a large cohort of PTC cases. In order to decrease unnecessary morbidity associated with over diagnosing PTC patient with good prognosis, we used TCGA data to develop a gene expression signature to distinguish between patients with good and poor prognosis. We selected a set of clinical phenotypes to define an ‘extreme poor’ prognosis group and an ‘extreme good’ prognosis group and developed a gene signature that characterized these.

**Results:**

We discovered a gene expression signature that distinguished the extreme good from extreme poor prognosis patients. Next, we applied this signature to the remaining intermediate risk patients, and show that they can be classified in clinically meaningful risk groups, characterized by established prognostic disease phenotypes. Analysis of the genes in the signature shows many known and novel genes involved in PTC prognosis.

**Conclusions:**

This work demonstrates that using a selection of clinical phenotypes and treatment variables, it is possible to develop a statistically useful and biologically meaningful gene signature of PTC prognosis, which may be developed as a biomarker to help prevent overdiagnosis.

**Electronic supplementary material:**

The online version of this article (doi:10.1186/s12885-016-2771-6) contains supplementary material, which is available to authorized users.

## Background

Papillary thyroid carcinoma (PTC) is not only the most common form of thyroid cancer; its incidence has been increasing faster than any other cancer type in the US [1–3]. The long-term prognosis of PTC is generally excellent. This rising incidence of PTC has been attributed, at least in part, to increased detection due to the rise and popularity of neck imaging studies [1, 2] The thyroid cancer prevalence rate in autopsy series around the world ranges from 6 to 36 % [4]. Most PTC patients are treated with surgery, radioactive iodine therapy, and thyroid hormone suppression; for most patients, this represents extreme overtreatment, as PTC has very low mortality with less than 1 % of cases succumbing from the disease [3]. A diagnosis and associated treatment of PTC carries significant financial and psychological burdens [5–10]. Treatment with radioactive iodine has been shown to clinically benefit only the patients with higher stages of disease, whereas its usefulness in lower stage patients, who constitute the vast majority of patients, has been debated. Given the serious potential side effects associated with radioactive iodine, as well as the excellent prognosis of patients with small tumors and no distant metastases at the time of presentation, the American Thyroid Association has recommended to consider radioiodine therapy only in patients with intermediate or high risk features on pathology. However, distinguishing these patients from the lowest risk patients can often be challenging. Biomarkers that distinguish good and poor prognosis patients would be very beneficial in guiding aggressiveness of treatment [11].

Molecularly, PTCs have few somatic alterations. They are mainly driven by mutations in the MAPK-pathway including NRAS, HRAS, KRAS and BRAF, and mutations in the PI3K-AKT signaling pathway [12]. Some of these mutations have been associated with either ionizing radiation or chemical mutagenesis. Recently, The Cancer Genome Atlas (TCGA) reported on the genomic landscape of PTC in 496 cases [13]. TCGA confirmed known drivers and also identified novel driver alterations, significantly reducing the fraction of PTC with unknown oncogenic events. The TCGA study identified two meta-clusters based on a BRAF-RAS signature dichotomizing PTC in BRAF-like and RAS-like subtypes.

The existing prognostic factors such as age at the time of diagnosis, the size of the tumor, extension into surrounding tissues, lymph node involvement, or distant metastasis help differentiate PTC patients into low and high risk [14]. However, the challenge for PTC is that these prognostic factors do not always allow the clinician to predict which “middle-risk” patients will have good vs. bad prognosis. Currently, there are no clear biomarkers to assist with prognostication. More specifically, there are no clear biomarkers that separate aggressive PTC from lesions that stay indolent for years. This has created an increasing challenge to study PTC prognosis due to the challenge of collecting long tumor follow-up data for biomarker discovery.

In this report, we take advantage of the large collection of genomic data collected in the TCGA cohort in combination with clinical data on treatment. We report that a gene expression signature exists with the potential to characterize low-risk disease. These results may lead to biomarkers that can change the management of low-risk disease leading to improvements in patient quality of life and reduced financial burdens [15].

## Methods

### Defining prognosis groups

Limited follow-up data was collected for the TCGA cohort and we used a collection of clinical phenotypes to define an ‘extreme poor’ prognosis group and an ‘extreme good’ prognosis group, based on features described in the Revised American Thyroid Association Management Guidelines for Patients with Thyroid Nodules and Differentiated Thyroid Cancer [16]. The remaining patients (74 %) are classified as ‘intermediate’ prognosis and are the cases where there is the highest clinical need to subdivide patients into finer categories of prognosis. For the extreme poor prognosis group, we included patients that had either one of the following seven characteristics: the patient died of thyroid cancer, the presence of distant metastases based on AJCC staging, persistent loco-regional or distant disease determined based on a person’s condition within 3 months of initial treatment, treatment with adjuvant drugs, treatment with IMRT and patients with a new tumor event after initial treatment. The extreme good prognosis group was defined as stage 1 patients without nodal involvement and absence of all of the poor prognosis characteristics used to define the extreme poor group. To compare our classification system with the MACIS score, MACIS scores for each patient were retrieved from the Additional files 1 and 2 section of the TCGA report [13].

### Molecular data processing

Preprocessed TCGA gene expression data (generated by RNA sequencing), DNA copy number data (generated by microarray technology), mutation data (generated by exome sequencing) and PARADIGM pathway activity data, were downloaded using the Firehose pipeline (version 2014071500 for gene expression and version 2014041600 for all other data sets) [5]. Preprocessing for these data sets was done according to the Firehose TCGA pipelines described elsewhere [5]. Additional preprocessing of this data set was done as follows: For the gene expression data, genes and patients with more than 10 % missing values were removed. All remaining missing values were estimated using KNN impute [17]. TCGA data were generated in batches, creating a batch effect for most data sets. Batch correction was done using Combat [18]. Significantly mutated genes were extracted from the mutation data using MutSig CV [19].

### Identifying gene expression signatures

We used the gene expression data to develop a prognostic classifier for thyroid cancer. We first selected the top 30 % most varying genes using the mean absolute deviation statistic, and subsequently used the z-score transformation for all genes so they have zero mean and unit variance. We used Significance Analysis of Microarrays (SAM) as previous described [20], to identify a gene expression signature that reflects prognosis based on genes that are differentially expressed between extreme prognostic groups. We selected the delta threshold such that the FDR was <0.05, and used 100 permutations.

### Comparison with adjacent normal tissue

The non-parametric Wilcoxon rank sum tests were used to test for significance of differences in expression between tumor and normal tissue, of genes identified as significantly associated with prognosis by SAM analysis.

### Evaluating the robustness of gene expression signatures

We tested the robustness of the gene expression signature by removing all patients defined by one of the seven poor prognosis characteristics from the previously defined group of extreme poor prognosis patients one at a time, and rebuilding the SAM signature using all remaining extreme poor and extreme good prognosis patients, and repeated this for each of the seven variables. We investigated the stability of the genes in the signature by counting the overlap of the genes in each of the seven analyses.

### Functional gene set enrichment analysis

Functional gene set enrichment (GSE) analysis was carried out using the GSE tools MSigDB [21] and Enrichr [22], selecting all gene-set libraries for comparison with the input prognostic gene-set. These included thousands of gene-sets from multiple databases, annotated to diverse disease and biological states and functions, as well as common regulatory mechanisms and motifs, identified by microarray experiments, data mining and curation of published data and knowledge. The prognostic gene-list was also compared with relevant gene-sets from additional sources, including a list of 861 known tumor suppressor genes from the TSgene database [23], a list of genes displaying bivalent epigenetic marks in embryonic stem cells [24], and a list of genes that are consistently deregulated in thyroid cancer, identified by meta-analysis [25]. Significance of overlap with these gene-sets was carried out using the hypergeometric test.

### Developing a supervised predictor of prognosis

Next, we used Prediction Analysis of Microarrays (PAM) to develop a parsimonious supervised prognostic gene signature [26]. PAM analysis uses a nearest shrunken centroids machine learning method that predicts the class (good/poor prognosis) based on the squared Euclidean distance of the gene expression profile for that sample to the centroids of known extreme good and poor prognosis patient groups. Shrinkage is used to select the optimum number of genes for class prediction. This means that the model will select only a subset of genes to develop the centroids.

We first used PAM in combination with 10-fold cross validation to determine the ability of the gene expression data to predict prognosis within extreme prognosis patients. For each fold of cross validation, the PAM model was trained on 90 % of patients and assigned class probability for good prognosis to the each of the remaining 10 % of patients based on the distance of the patient to its closest centroid. We used the Area under the ROC curve (AUC) to evaluate the performance of the model in accurately predicting the prognostic class of patients.

### Application of the supervised predictor to intermediate prognosis individuals

We applied this prognostic gene expression signature to intermediate risk patients to classify them into either good or poor prognosis groups, using gene expression data for the top 20 % most varying genes (i.e., with the highest mean absolute deviation). To classify a new sample, its distance is calculated to each of the centroids by using the weights as an inner product, and the sample is classified to its closest centroid. We only used classification results when probabilities were >60 % or <40 %. Low confidence assignments for the remaining borderline individuals were excluded from further analyses.

### Evaluating the robustness of the classifier

We tested the robustness of the PAM classifier to split the patients into good or poor prognosis groups by removing patients featuring one of the seven poor prognosis characteristics from the previously defined group of extreme poor prognosis patients, and rebuilding the PAM classifier using all remaining extreme poor and extreme good prognosis patients. We investigated the stability of the genes in the classifier and investigated the classification assignments of the left-out group. In addition, we classified the intermediate prognosis thyroid cancer cases into a ‘intermediate-poor’ prognosis and ‘intermediate-good’ prognosis groups, and reported the distribution of mutations, clinical stage, nodal involvement, extra-thyroid extensions and histological subtypes, between these groups.

### Testing for pathological and confounding clinical factors

Significance of differences in distribution of categorical factors such as gender and nodal involvement was tested using Pearson’s chi-squared with Yates correction for small numbers of samples within some categories. Student’s *T*-test was used to test difference in age between prognostic groups.

## Results

### Identification of a thyroid prognosis gene expression signature

**Fig. 1:**
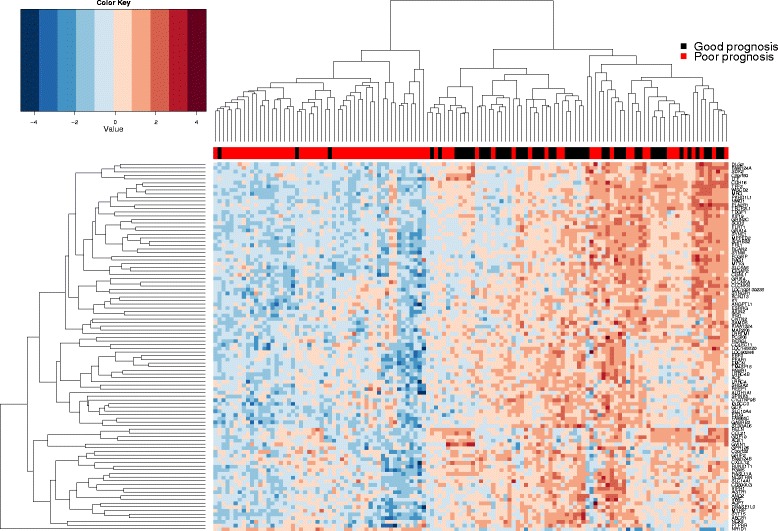
Association of gene expression with prognosis within the TCGA papillary thyroid cancer study. Heatmap showing expression of the top 100 genes that were most differentailly regulated between papillary thyroid cancer patients of good (*n* = 51) and poor (*n* = 79) prognosis, tested using significance analysis of microarrays (SAM) analysis. Genes and samples are arranged by linkage distace, using unsupervised hierarchical clustering of average expression across samples and genes, respectively, as illustrated by dendrograms. Good and poor prognosis patients are represented by *red* and *black* squares within the sidebar

We focused on extreme phenotypes to develop a prognostic expression signature for PTC. Using the prognostic clinical characteristics defined in the methods, we identified 79 extreme poor and 51 extreme good prognosis cases out of 494 cases in the TCGA thyroid cancer cohort, for which RNA gene expression data were available (Fig. 1). Prognosis groups did not differ significantly in distributions of histological subtypes or demographic factors for which data were available, including age, gender or race (Additional file 1: Table S1). The poor prognosis group had a significantly higher MACIS score (a clinically used prognostic score based on clinical features, including presence of distant metastases, patient age, completeness of resection, local invasion and tumor Size) (*p* = 4.28e-07, Additional file 2: Figure S1). We first used univariate analysis to identify if gene expression is discriminatory of these extreme prognosis thyroid cancer cases. Using SAM we identified ten genes upregulated in extreme poor prognosis patients and 791 genes downregulated in extreme poor prognosis patients (Fig. 1, Additional file 1: Table S2).

### Differential expression between normal and tumor tissue

To test if the signature genes are also differentially expressed compared to adjacent normal tissue, we investigated whether there was a significant difference in gene expression between all tumor (*n* = 501) and adjacent normal tissue (*n* = 58) samples within the PTC study. Of 791 genes downregulated in the extreme poor prognosis group relative to the extreme good prognosis group (according to SAM analysis), 674 (85 %) were significantly differentially regulated between tumor and adjacent normal tissue. Of these, 611 (91 %) were downregulated in tumor, whereas 63 (9 %) were upregulated in tumors. All ten genes upregulated in the extreme poor prognosis group according to SAM analysis were also significantly upregulated in tumor versus normal tissue (Additional file 1: Table S2).

**Fig. 2:**
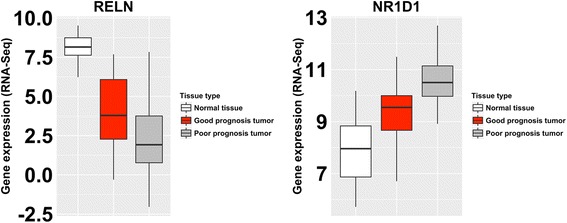
Differential expression of genes between normal tissue, good prognosis and poor prognosis patient groups within the TCGA thyroid cancer study. Representative examples of genes that were identified as differentially expressed between good and poor prognosis tumors, and which were also differentially expressed between tumor and normal tissue. These genes displayed step-wise changes of expression between normal tissue, good prognosis tumors and poor prognosis tumors, which may be indicitive of incremented deregulation associated with advancing disease

For most genes within this signature there was a an incremental pattern of expression from normal tissue, to extreme good prognosis patient tumor, to extreme poor prognosis patient tumor, such that there were significant linear association of expression in this direction (Additional file 1: Table S3, Fig. 2).

### Robustness of gene signature

To narrow down the gene signature, we investigated the robustness of this signature by removing patients defined by each of the seven poor prognosis clinical characteristics, one at a time, and calculating the gene overlap. None of the genes upregulated in extreme poor prognostic patients remained consistently upregulated when each prognostic characteristic was left out. One of these genes, *NR1D1* was the most consistently upregulated gene, upregulated when four of seven groups were removed. For the downregulated genes, we identified 109 genes that were robust to leaving out each of the poor prognosis characteristics (Additional file 1: Table S4), 100 (92 %) of which were also significantly downregulated in tumor compared with normal adjacent tissue.

### Functional enrichment analysis

The prognostic gene signature of 109 genes most significantly overlapped with a list of genes that were downregulated in PTC compared to normal tissue in a previous study [27], with 27 overlapping genes (*q* = 1.75 e^−34^, Additional file 1: Table S5). Also significantly overlapping was a set of 17 genes downregulated in basal subtype breast cancer [28]. Among gene ontology (GO) terms within MSigDB, genes downregulated in poor prognosis were enriched for the genes with ‘bivalent’ promoters, i.e. genes with CpG-dense promoters bearing both the activating H3K4me3 and the repressive H3K27me3 histone marks, in brain [29]. To confirm this, the prognostic gene signature was compared with a list of all known bivalent genes from the BGDB database [24]. Of 109 genes within the prognostic signature, 53 were bivalent (*p* = 1.96e-12). There were no other highly enriched gene ontology (GO) terms or overlaps with gene-sets representing specific biological mechanisms such as transcription factor binding sites or organelle functions; therefore, the prognostic genes signature is not likely related to a single tumor event or characteristic, but reflects diverse abnormalities in multiple cancer pathways. Using the Enrichr enrichment analysis tool, the gene-set with which the 109 poor prognosis genes most significantly overlapped was an independent list of genes that were deregulated in PTC, derived from an independent study (*q*-value = 1.052e-14), with 21 overlapping genes [30]. Next, the 109 poor prognosis genes were compared with known tumor suppressor genes [23]. Nine listed tumor suppressor genes (*LTF*, *RASL11A*, *SYNM*, *IQGAP2*, *AXIN2*, *SLC5A8*, *LIFR*, *IGFBPL1*, *ZNF366*) were among our poor-prognosis genes, a significant enrichment (*p* = 0.04). Finally, our gene list was compared to a gene-set of 39 genes consistently deregulated in thyroid cancer, identified by meta-analysis of multiple studies [25]. Of these, *TFF3*, *DIO1* and *ITPR1* were overlapping.

### A supervised predictor accurately classifies prognosis

**Fig. 3:**
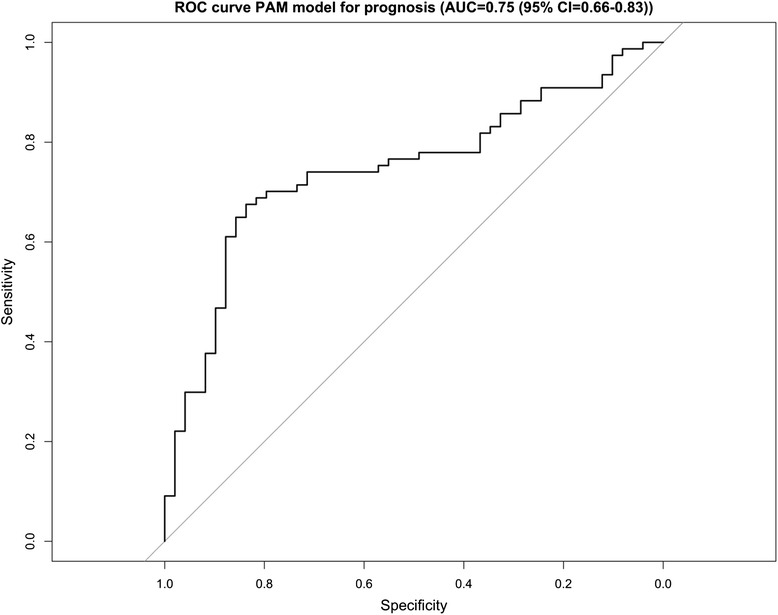
Performance of a gene-expression based supervised predictor in classifying prognosis. ROC curve illustratrating the performance of a gene-expression based supervised classifier in correctly predicting the prognostic group (good or poor prognosis) to which each patient belongs, over 10 rounds of cross-validation. The classifier was determined using Prediction of Microarray (PAM) analysis, and was limited to 100 genes, which were differentially expressed between good and poor prognosis patients

Next, we estimated the classification performance of a supervised classifier to predict prognosis using the extreme poor and good prognosis patients. We used the PAM classifier in combination with 10-fold cross validation and limited the classifier to maximum 100 genes. This resulted in an AUC of 0.75 (95 % CI 0.67–0.84, Fig. 3), indicating that a gene expression signature exists that is predictive of prognosis.

### Distinguishing thyroid cancer cases with intermediate prognosis

**Fig. 4:**
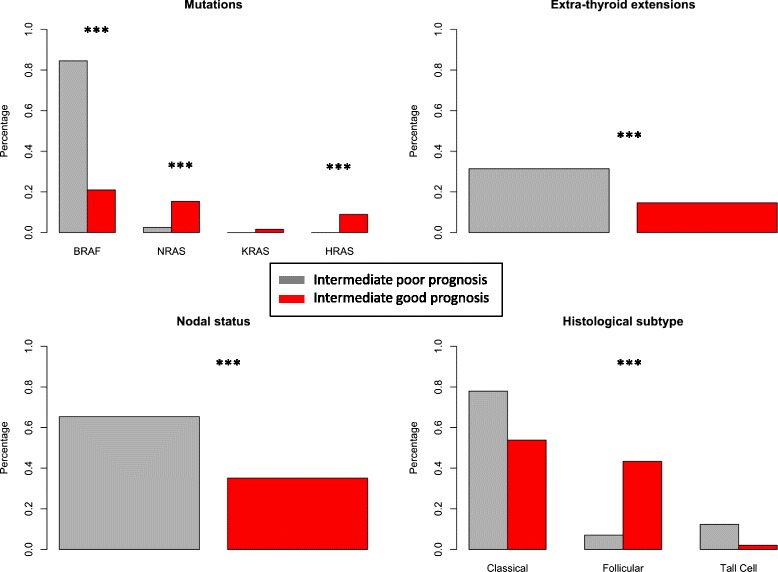
Enrichment of clinicopathological prognositic features of papillary thryroid cancer within intermediate prognosis patients classified as good and poor prognosis by a Prediction of Microarrays (PAM) model. Intermediate prognosis patients (*n* = 378) were classified as either good (*n* = 111) or poor prognosis (*n* = 195) using the PAM model, which was trained using the gene signature of differential expression between extreme good prognosis (*n* = 51) and extreme poor prognosis (*n* = 79) patients. Within the poor prognosis group there was a higher percentage of patients with *BRAF* mutations, nodal involvement, extra-thyroid extension, and the aggressive Tall cell histological subtype, but a lower percentage of patients with NRAS and HRAS mutations and the well-differentiated follicular histological subtype. *** Chi -squared *p*-value < 0.001

As a preliminary validation for the expression PAM classifier, and to test its ability to predict prognosis/disease outcome in intermediate prognosis individuals (the group for which prognostic classification is required), we built a PAM model on the complete data set of extreme prognosis cases and classified the remaining intermediate prognosis PTC cases in two groups: intermediate-poor prognosis and intermediate-good prognosis. We then compared the distribution of key mutations and pathological variables relevant to prognosis between these groups. Out of 378 intermediate prognosis patients, PAM analysis assigned 306 patients to either intermediate-good or intermediate-poor prognosis groups with ‘high confidence’ probabilities of >60 % or <40 %, respectively. Of the 306 intermediate prognosis patients with high-confidence assignments, 111 (36 %) were classified as intermediate-good prognosis and 195 (64 %) were classified as intermediate-poor prognosis. The intermediate-poor prognosis group had higher nodal involvement, a tendency towards extra-thyroid extension and is highly enriched for BRAF mutations compared to the good prognosis group. The intermediate-good prognosis group had a mixed RAS-BRAF mutation composition, with significantly higher incidence of HRAS and NRAS mutations compared with the poor prognosis group (Table 1). There was a significantly higher incidence of the well-differentiated follicular cell histological subtype and a depletion of the more aggressive tall-cell subtype within the intermediate-good prognosis group (Fig. 4). There were no significant differences in distributions of age, gender or race between prognosis groups. There was no difference in MACIS score between intermediate-poor (*n* = 189) and intermediate-good prognosis groups (*n* = 146) (*p* = 0.2, Additional file 2: Figure S1). Similar enrichments in the intermediate prognosis group were found when all samples (including the 72 individuals with posterior probabilities between 40 and 60 %) were analyzed (Additional file 1: Table S6).

**Table 1:** Distribution of clinicopathologic and demographic factors in intermediate risk patients classified as good or poor prognosis by PAM model

Factor			Poor prognosis % (n)	Good Prognosis % (n)	*P*-value^a^ (different frequency between prognostic groups)
Extra-thyroid extension		N total	144	130	0.001
		With extentions	32 % (47)	15 % (19)	
Nodal involvement		N total	127	114	<0.001
		with nodal involvement	65 % (83)	35 % (40)	
Clinical stage		N total	144	135	0.08
		Stage 1	22 % (32)	22 % (30)	
		Stage 2	40 % (58)	52 % (70)	
		Stage 3	38 % (54)	26 % (35)	
Histological subtype		N total	179	142	<0.001
		Classical	80 (144)	54 (77)	
		Follicular	5 (9)	44 (62)	
		Tall cell	15 (26)	2 (3)	
Age at diagnosis		N total	184	143	0.41
		less than 45 years	50 (92)	45 (64)	
Gender		N total	184	143	0.7834
		Female	72 % (132)	76 % (108)	
		Male	28 % (52)	24 % (35)	
Race		N total	154	55	0.544
		Asian	11 % (17)	14 % (8)	
		Black/African American (n)	7 % (11)	4 % (2)	
		White	82 % (126)	82 % (45)	
Mutations	BRAF	N total	162	124	<0.001
		With mutations	85 % (137)	21 % (26)	
	HRAS	N total	162	124	<0.001
		With mutations	0 %	9 % (11)	
	KRAS	N total	162	124	0.36
		With mutations	0 %	2 % (2)	
	NRAS	N total	162	124	<0.001
		With mutations	2 % (4)	15 % (19)	

^a^(Pearson chi-squared (categorical variables), or Student’s *T*-test (continous variables)

### Robustness analysis of the supervised predictor

We investigated the robustness of the PAM supervised predictor, i.e. its performance in predicting prognosis when each poor prognostic clinical characteristic was excluded. Extreme poor prognosis patients assigned to each of the seven poor prognostic factors were excluded, one group at a time. For each left-out group, a PAM predictor was trained using the remaining extreme prognosis samples, and the performance of the model in accurately predicting prognosis for left-out individuals was assessed. For all but one of the left out groups, between 11 and 43 % of the left out samples were classified as good prognosis without including them for training (Additional file 2: Figure S2, Additional file 1: Table S7). Additionally, we predicted prognosis for the intermediate prognosis thyroid cancer cases and investigated the enrichment of mutations, stage, nodal status, extensions and histological subtypes in the cases classified as intermediate-good or intermediate-poor prognosis. This confirmed the previously reported profile of poor prognosis patients characterized as BRAF mutated with high stage, lymph node invasion, as well as enrichment for the tall cell subtype and depletion of the follicular subtype, even when removing one of the seven poor prognosis characteristics (Additional file 1: Table S7). When examining the genes defining these supervised predictors, we identified 56 genes that are selected in at least 6 out of seven left out analyses (Additional file 1: Table S8). This signature included five genes upregulated in poor prognosis patients, including *NR1D1*, a gene overlapping with the thyroid receptor *THRA*, the oncogene *AREG*, and 51 genes downregulated in poor prognosis patients, including many of the poor prognosis genes that were overlapping between our study and gene-sets downregulated in thyroid cancer identified by GSE analysis, TSgene tumor suppressor genes (*LTF*, *RASL11A*, *IQGAP2*, *SYNM*), and members of cancer-related pathways, such as *FZD9*, a member of the Wnt signaling pathway, *MAP2K6*, a MAP kinase, *IGFBPL1*, a regulator of insulin-like growth factor signaling, and *LRRC4* and *LRRC4B* involved in cell proliferation, migration and angiogenesis.

## Discussion

We used a large cohort of PTC patients from the TCGA project with detailed but heterogeneous clinical data to determine a prognostic signature to separate good from poor prognosis focusing on intermediate risk PTC. We used extreme phenotypes based on a homogeneous definition of good prognostic PTC cases and seven heterogeneous clinical characteristics determining poor prognostic PTC. We discovered genomic signatures that potentially allow distinguishing good from poor prognosis. Due to the indolent nature of PTC, most patients do not die from PTC and long-term follow-up is often not available, as is the case in TCGA. However, our work shows that using clinical data capturing treatment-related variables, it is possible to develop genomic signatures of prognosis consisting of known tumor suppressor genes, genes known to be deregulated in PTC, and genes with specific roles in thyroid function. As expected, the commonly used MACIS prognostic score was higher in extreme poor prognosis patients defined by our classification system, as we use some of the same clinical data as used by the MACIS score. However, MACIS score did not differ between intermediate risk patients predicted as poor versus good prognosis by our prognostic signature, indicating that our prognostic signature predicts prognosis based on information not picked up by the MACIS score. This is likely because our classifier does not rely on the clinical features used by the MACIS score, which are mainly found within the more extreme PTC cases of more obvious prognosis. Instead, our classifier captures ‘hidden’ prognostic information within the tumor gene signature, which can be detected in intermediate risk patients for which prognosis can rarely be predicted using classification systems based on clinical information alone. Over 90 % of the genes deregulated in poor prognosis patients relative to good prognosis patients were also significantly deregulated in PTC relative to normal adjacent tissue, in the same direction, so that good prognosis patients displayed expression levels of prognostic genes that were intermediate between normal tissue and poor prognosis patients. This indicates more advanced gene deregulation in poor prognosis individuals, and indicates that the relationship between these genes and cancer prognosis is approximately linear. As the poor and good prognosis patient groups had similar distributions of histological subtypes and demographic factors for which data are available, the differentially expressed genes are unlikely to reflect different histological subtypes or confounding factors. Moreover, the striking enrichment within the poor prognosis gene-set of genes downregulated in papillary PTC indicates that our poor-prognosis genes set is highly specific to PTC. The genes overlapping between our poor prognostic signature and previously reported thyroid cancer signatures (Additional file 1: Table S5) provides a list of genes that are reproducibly downregulated in thyroid cancer, which are also associated with poor prognosis, and represent promising potential biomarkers.

Some poor prognosis genes identified were specific to thyroid function. DIO1, downregulated in poor prognosis patients in this study and a meta-analysis [25], is required for both activation and degradation of thyroid hormone. The most consistently upregulated gene in poor-prognosis patients, NR1D1, is antisense to, and overlapping with the thyroid receptor gene THRA, and these two genes form a *cis*-natural antisense pair in tail-to-tail orientation (with 3’ ends overlapping), so that THRA transcription is impeded by NR1D1 transcription [31]. This was supported by a modest, but significant negative correlation between NS1D1 and THRA expression within the TCGA PTC study in primary tumors (cor = −0.09, *p* = 0.04, *n* = 501), though not within a smaller set of normal adjacent tissue samples (cor = −0.16, *p* = 0.24, *n* = 58) (Additional file 2: Figure S3). While THRA is frequently mutated in thyroid cancer, little has been reported about the role of THRA in cancer [32].

Next, there was a strong enrichment for genes downregulated within the poor prognosis group, relative to those patients with a good prognosis. Many of these genes were downregulated in good prognosis tumors relative to normal tissue, and further downregulated in poor prognosis tumors, indicating that incremental loss of expression may promote cancer progression. Alternatively, these genes may represent markers of more aggressive PTC subtypes. Given the apparent enrichment of genes downregulated in cancer, it is tempting to speculate that a common mechanism of gene repression may contribute to poor prognosis in PTC. One such mechanism is aberrant DNA methylation of tumor suppressor gene promoters in cancer, which commonly occurs at genes that display bivalent epigenetic signatures in embryonic stem cells [33]. There was a strong enrichment for bivalent genes within our prognostic signature; therefore, epigenetic silencing of bivalent genes may represent a common mechanism accounting for the enrichment of downregulated genes in cancer associated with poor prognosis. Supporting this is the identification of multiple genes downregulated in poor prognosis patients that are reported to be epigenetically silenced in cancer, such as SYNM [34] and IGFBPL1 [35]. Many bivalent genes play key roles in maintaining cellular differentiation, and their silencing in cancer is thought to promote de-differentiation and pluripotency.

Some the poor prognosis genes identified, such as TFF3 and CDH16, represent well-established thyroid cancer markers. TFF3 has previously been proposed as a potential biomarker to discriminate between benign from malignant PTC [25, 36]. CDH16 is specifically expressed in kidney and thyroid, playing a role in thyroid differentiation and epithelial-mesenchymal transition, and strongly downregulated across PTC subtypes [37]. CLCNKB, a chloride channel, is downregulated in malignant papillary PTC versus benign disease [38]; however, the oncogenic role of CLCNKB is unknown. PLA2R1 is downregulated in malignant PTC versus benign disease [38], and appears to suppress tumorigenesis by activating the tyrosine kinase JAK2 [39]. Two ‘RAS-like’ genes of unknown function, RASL11A and RERGL, as well as RASF9, were downregulated in poor prognosis patients. Given the important etiological role of RAS signaling in PTC, exploration of the function of these RAS-like genes in prognosis is warranted. Other notable genes of unknown function within this list were WSCD2 and IQGAP2, which have previously been reported as downregulated in thyroid cancer [25].

Some genes within poor-prognosis PTC patients may not be specific to PTC, as some are known tumor suppressor genes and prognostic markers within other cancers. Both LRRC4 and the closely related LRRC4B were downregulated in poor prognosis patients. LRRC4 is a tumor suppressor gene in glioblastoma, apparently regulating ERK/AKT/NF-kB signaling [40]. That these genes may also be tumor-suppressor genes in PTC is a novel and interesting finding, as expression of LRRC4 is thought to be restricted to nervous tissue. Epigenetic silencing of IGFBPL1, a member of the insulin-like growth factor binding protein, is associated with nodal involvement and poorer outcome in breast cancer [35]. Epigenetic silencing of the Synemin tumor suppressor gene (SYNM) in breast cancer is associated with poorer survival, lymph node involvement and advanced tumor grade [34] MAP2K6 was a member of a four gene-panel used to predict prognosis in bladder cancer [41].

In addition, our results show that although the BRAF-RAS signature is enriched in the prognostic signature it is not an exact separator of prognosis. Although mutations in RAS genes are only enriched in good prognosis cases and BRAF mutations occur mostly in the poor prognosis cases, there is still a significant group of good prognosis cases with BRAF mutations. We also investigated developing prognostic signatures using DNA copy number, DNA methylation and microRNA data, but these models were less predictive of prognosis than gene expression signatures (data not shown). This is not surprising, as epigenetic mechanisms and copy number variation likely influence disease outcome through alteration of gene expression, therefore measurement of expression itself may be more directly related to disease outcomes.

## Conclusions

There is a pressing clinical need for prognostic biomarkers to direct therapy and treatment planning for patients presenting with PTC. Given the precipitous rise in this disease, robust clinical indicators may help reduce the potential consequences of overtreatment for patients with this mostly indolent disease. Our identification of a prognostic signature for PTC provides proof of concept that gene expression patterns can be used to identify patients who may otherwise be subject to overdiagnosis. This work may provide a rational first step towards identifying a prognostic test that can help clinicians to tailor therapy in patients with good prognosis and intensify management of patients with poor prognosis. The analytical methods we adopted provided an alternative to standard survival analysis to identify genes associated with prognosis, due to the small number of deaths from PTC. Unfortunately, this analytical method does not entail adjustment for potential confounding factors that may influence patient prognosis such as age or different treatments. The key limitation of this study, however, is that we were unable to validate our prognostic gene signature in independent patient cohort, due to lack of existing data or samples with clinical annotation. Our work motivates the collection of long-term clinical follow-up data to further develop, refine and validate prognostic signatures for PTC. Such a signature may be developed as a clinically applicable biomarker using technologies such as the NanoString nCounter (The platform used for the commercially available PAM50 breast cancer test [42]) to routinely measure expression of hundreds of genes. Moreover, it will also be important to determine whether this signature is detectable in ultrasound-guided fine needle aspirate (FNA) biopsies that are collected routinely for examination of thyroid nodules to detect cancer. Identification of patients with good prognosis PTC at this stage may allow them to avoid unnecessary surgery.

## Additional files

Additional file 1: Table S1. Demographic factors in extreme good and extreme poor prognosis patient groups (in samples for which data was available). **Table S2.** Genes deregulated in extreme poor prognosis PTC patients relative to extreme good prognosis patients. **Table S3.** Differential expression of prognostic genes in normal tissue, extreme good prognosis cancers and extreme poor prognosis cancers. **Table S4.** Genes consistently upregulated or downregulated [1] in extreme poor prognosis patients in leave-one-out cross validation. **Table S5.** Genes downregulated in poor-prognosis PTC & overlapping with published (referenced) gene-sets. **Table S6.** Distribution of clinicopathologic and demographic factors in intermediate risk patients classified as good or poor prognosis by PAM model (All samples). **Table S7.** Distribution of clinical characteristics of intermediate good and intermediate poor prognosis groups in leave-one-out cross validation. **Table S8.** Genes used within the PAM classifier, and presence [1] or absence (0) of each gene within classifier, when patients representing each of 6 poor prognostic factors are left out. (PDF 1097 kb) Additional file 2: Figure S1. MACIS score in extreme prognsos and intermediate prognosis patient groups: MACIS score (based on the presence of distant Metastases, patient Age, Completeness of tumor resection, presence of local Invasion, and tumor Size) was higher in poor prognosis patients (*n* = 77) than good prognosis patients (*n* = 50) within the Extreme prognosis patient (training set) patient group (*p* = 4.28e-07). However, there was no difference in MACIS score between patients predicted as poor (*n* = 189) and good prognosis (*n* = 146) witin the medium prognosis (test set) patient group (*p* = 0.2). This indicates that the MACIS score, indicating that the MACIS score does not have ability to predict patient prognosis as determined by our gene expression classifier. **Figure S2.** Checking the robustness of the PAM model gene signature: Leaving out one of 7 extreme prognosis patient groups in each round, and using the remaining 6 patient groups to train a Prediction Analysis of Microarrays (PAM) model, the performance of the PAM model in correctly classifying patients within the left-out group to either the good or poor prognosis groups, was tested. ROC curves illustrate the performances of the models for each left out group. Left out groups represent extreme poor prognosis patients, each group associated with a specific poor prognostic clinical factor. **Figure S3.** Negative correlation between *NR1D1* and the overlapping thyroid hormone receptor gene *THRA*. There was a significant Pearson correlation of expression of NR1D1 and THRA (measured using RNA-Seq) in tumor (cor = −0.09, *p* = 0.04, *n* = 501), but not within a smaller set of normal adjacent tissue samples (cor = −0.16, *p* = 0.24, *n* = 58). NR1D1 was the gene most strongly upregulated in poor prognosis PTC patients relative to good prognosis patients, and may influence thyroid cancer through downregulation of THRA. (PPTX 1109 kb)

## Abbreviations

AJCC: American joint committee on cancer
AUC: Area under the (receiver operating) curve
GO: Gene ontology
GSE: Gene set enrichment
PAM: Prediction of microarrays
PTC: Papillary thyroid cancer
SAM: Significance of microarrays
TCGA: The Cancer Genome Atlas
