# Supplementary material for: Development of prognostic signatures for intermediate-risk papillary thyroid cancer
Source: BMC Cancer. 2016 Sep 15;16:736. doi: 10.1186/s12885-016-2771-6 (PMC5025616; doi:10.1186/s12885-016-2771-6)
Supplement: Additional file 1: Table S1. — Demographic factors in extreme good and extreme poor prognosis patient groups (in samples for which data was available). Table S2. Genes deregulated in extreme poor prognosis PTC patients relative to extreme good prognosis patients. Table S3. Differential expression of prognostic genes in normal tissue, extreme good prognosis cancers and extreme poor prognosis cancers. Table S4. Genes consistently upregulated or downregulated [1] in extreme poor prognosis patients in leave-one-out cross validation. Table S5. Genes downregulated in poor-prognosis PTC & overlapping with published (referenced) gene-sets. Table S6. Distribution of clinicopathologic and demographic factors in intermediate risk patients classified as good or poor prognosis by PAM model (All samples). Table S7. Distribution of clinical characteristics of intermediate good and intermediate poor prognosis groups in leave-one-out cross validation. Table S8. Genes used within the PAM classifier, and presence [1] or absence (0) of each gene within classifier, when patients representing each of 6 poor prognostic factors are left out. (PDF 1097 kb) [file 12885_2016_2771_MOESM1_ESM.pdf]

Supplementary table 1: Demographic factors in extreme good and an extreme poor prognosis patient groups (in samples for which data was available).

Demographic factor	Level	Extreme Poor prognosis	Extreme good prognosis
**Total**		79	51
**Race**	American Indian or Alaska Native	1	
	Asian	6	
	Black/African American	8	7
	White	57	42
**Gender**	Female	54	42
	Male	25	9
**Histological subtype**	Classical/usual	65	36
	Follicular (>= 99% follicular patterned)	10	12
	Tall Cell (>= 50% tall cell features)	3	2
**Age**	Age (n <45 years)	28	32

Supplementary table 2: Genes deregulated in extremem poor prognosis PTC patients relative to extreme good prognosis patients

Gene Name	Fold Change	Q-value (%)	Normal vs. Tumor P-value	Average Expression Difference Normal Minus Tumor
NR1D1	1.85	0.00	<0.001	-2.13
LIMS3	1.72	1.28	<0.001	-0.58
IQGAP3	1.72	1.28	<0.001	-2.49
SNX22	1.70	1.28	<0.001	-1.02
FSTL3	1.69	1.28	<0.001	-1.29
AREG	1.68	1.28	0.001	-0.92
CELF4	1.67	1.28	<0.001	-2.8
C1orf51	1.64	2.22	<0.001	-0.88
EPHA10	1.63	2.22	<0.001	-3.68
FLJ23867	1.59	4.70	<0.001	-1.85
MGAT5B	0.79	4.70	0.145	0.29
ABCA8	0.79	4.70	<0.001	1.73
NPR1	0.79	4.70	<0.001	0.52
CECR2	0.79	4.70	<0.001	3.29
CNTNAP2	0.79	4.70	<0.001	1.74
FAM83B	0.79	4.70	<0.001	1.09
FXYD1	0.79	4.70	<0.001	2.83
ROR2	0.79	4.70	<0.001	2.47
TBXA2R	0.79	4.70	<0.001	-0.71
MRGPRF	0.79	4.70	<0.001	1.32
SLC38A11	0.78	4.70	<0.001	-1.47
WDR72	0.78	4.70	<0.001	2.56
CCDC151	0.78	4.70	0.001	0.55
ATP1A3	0.78	4.70	0.308	0.21
FOLR2	0.78	4.70	<0.001	-0.5
CTNND2	0.78	4.70	<0.001	1.43
MGC42105	0.78	4.70	<0.001	1.18
KIAA1324L	0.78	4.70	<0.001	0.98
NLGN3	0.78	4.70	<0.001	1.44
GLT8D2	0.78	4.70	<0.001	2.61
C1QTNF2	0.78	3.12	<0.001	1.29
RUNDC2A	0.78	4.70	<0.001	1.01
TXNDC3	0.78	4.70	<0.001	-0.83
POU2AF1	0.78	4.70	0.075	0.73
MGAM	0.78	4.70	0.008	-0.3
OSR1	0.78	4.70	<0.001	3.15
CD209	0.78	4.70	0.581	-0.07
MAB21L1	0.78	3.12	<0.001	1.55
LRP8	0.78	4.70	<0.001	0.95
CES3	0.78	4.70	0.231	-0.22
THSD7B	0.78	2.22	0.469	0.01
HGF	0.78	4.70	<0.001	1.58
PTGDS	0.78	3.12	<0.001	1.68
RGS18	0.78	4.70	0.818	0.08
COL6A6	0.78	4.70	<0.001	1.62
CSMD2	0.78	4.70	<0.001	-0.83
PVRL3	0.78	4.70	<0.001	0.97
C20orf151	0.78	4.70	<0.001	0.62
PRPH2	0.78	4.70	<0.001	1.37
CD4	0.78	4.70	<0.001	-0.59
SLC15A3	0.78	3.12	0.079	-0.21

GRAMD2	0.78	4.70	<0.001	2.11
BVES	0.78	3.12	<0.001	0.99
KCNK3	0.78	4.70	<0.001	0.51
C18orf34	0.78	4.70	<0.001	1.88
TEKT3	0.78	4.70	<0.001	1.69
TTC16	0.78	4.70	<0.001	1.1
GPR65	0.78	4.70	0.962	0.08
NQO1	0.78	4.70	0.806	-0.14
CNR1	0.78	4.70	0.009	0.5
MDS2	0.78	3.12	<0.001	1.31
KIF21B	0.78	4.70	0.002	0.72
CYTH4	0.78	4.70	0.202	-0.17
EDN3	0.78	3.12	<0.001	3.4
EPHB1	0.78	4.70	<0.001	2.63
PDE3B	0.77	4.70	0.001	0.65
RGS5	0.77	3.12	<0.001	-0.7
DCLK3	0.77	3.12	<0.001	-1.62
FLRT2	0.77	3.12	<0.001	1.61
DUOX2	0.77	3.12	0.799	0.01
LOC150197	0.77	4.70	<0.001	0.8
SIGLEC5	0.77	4.70	0.224	-0.18
TLL1	0.77	4.70	<0.001	0.76
AGAP2	0.77	3.12	0.792	0.14
XIST	0.77	4.70	0.943	0.03
LILRB1	0.77	4.70	0.003	-0.64
GDPD3	0.77	3.12	0.088	0.21
FRMPD2	0.77	4.70	<0.001	-1.26
SHBG	0.77	4.70	<0.001	0.88
SMTNL2	0.77	3.12	0.001	-0.56
MUSTN1	0.77	3.12	<0.001	1.11
ANKRD20A3	0.77	4.70	<0.001	1.95
FAM181B	0.77	4.70	<0.001	2.13
CDC14C	0.77	4.70	<0.001	1.49
CABP4	0.77	3.12	0.069	-0.23
EMR1	0.77	4.70	<0.001	-0.76
OR2W3	0.77	3.12	<0.001	1.57
WNT11	0.77	3.12	<0.001	3.04
GPR18	0.77	4.70	0.01	0.9
IL10RA	0.77	4.70	0.551	-0.01
GRAPL	0.77	3.12	0.001	0.52
CFP	0.77	3.12	0.216	-0.05
DNAH9	0.77	3.12	<0.001	1.33
TPH1	0.77	4.70	0.266	-0.25
TMEM63C	0.77	4.70	0.144	0.14
NHS	0.77	3.12	<0.001	1.54
SIGLEC1	0.77	3.12	<0.001	-0.6
ST6GAL1	0.77	4.70	<0.001	0.69
CEL	0.77	4.70	0.203	-0.14
DENND2A	0.77	3.12	<0.001	0.74
CD180	0.77	3.12	0.215	-0.14
TLR10	0.77	3.12	0.761	0.16
C1QTNF7	0.77	3.12	<0.001	2.66
FMOD	0.77	3.12	<0.001	1.87
FOXP2	0.77	3.12	<0.001	3.29
DBH	0.77	3.12	<0.001	0.79

SLC7A9	0.77	4.70	<0.001	1.26
EPHA3	0.77	3.12	<0.001	2.44
LOC387646	0.77	3.12	<0.001	1.56
EDARADD	0.77	3.12	<0.001	1.71
CT62	0.77	3.12	0.696	0.13
ZNF98	0.77	3.12	<0.001	0.9
CEBPA	0.77	3.12	0.001	0.43
ABCD2	0.77	3.12	<0.001	1.02
DES	0.77	4.70	<0.001	3.67
HCG2P7	0.77	4.70	<0.001	0.84
NCAM1	0.77	3.12	<0.001	2.82
JAKMIP3	0.77	3.12	<0.001	1.71
WFIKKN2	0.77	3.12	0.761	0.07
SLAMF6	0.77	3.12	<0.001	1.15
PPARGC1A	0.76	3.12	<0.001	2.81
PHKG1	0.76	4.70	<0.001	0.72
SPIB	0.76	3.12	0.028	1.11
GAPT	0.76	3.12	0.299	-0.11
CD8B	0.76	3.12	0.002	0.83
GFI1	0.76	3.12	<0.001	0.82
PKDCC	0.76	2.22	<0.001	1.53
CD79B	0.76	3.12	0.01	-0.18
PDE10A	0.76	2.22	<0.001	1.57
ZNF488	0.76	3.12	<0.001	0.84
DNALI1	0.76	3.12	<0.001	1.43
SLC6A16	0.76	3.12	<0.001	2.42
SOSTDC1	0.76	3.12	<0.001	3.12
HRH2	0.76	3.12	<0.001	1.12
F13A1	0.76	3.12	0.965	-0.01
HIGD1B	0.76	3.12	<0.001	-2.32
FERMT1	0.76	3.12	<0.001	2.2
FAM95B1	0.76	3.12	<0.001	1.78
CKMT2	0.76	3.12	0.489	0.09
CYP46A1	0.76	3.12	<0.001	1.02
ROPN1B	0.76	3.12	<0.001	2.19
SYBU	0.76	2.22	<0.001	0.98
TRIM58	0.76	3.12	<0.001	1.67
VIP	0.76	3.12	0.333	0.15
GATM	0.76	3.12	<0.001	1.57
RNF150	0.76	3.12	<0.001	2.62
MYOM3	0.76	3.12	<0.001	-2.1
AOC2	0.76	3.12	<0.001	0.58
SLA	0.76	3.12	<0.001	0.53
LOC728606	0.76	3.12	0.583	0.43
CRYBB1	0.76	2.22	0.001	-0.58
COL14A1	0.76	2.22	0.056	0.35
GNAL	0.76	3.12	<0.001	1.58
CHST8	0.76	3.12	<0.001	1.14
ADIG	0.76	3.12	<0.001	2.9
MGC16703	0.76	3.12	<0.001	0.61
BCL2L15	0.76	3.12	<0.001	0.67
ST8SIA1	0.76	3.12	<0.001	1.49
HSD17B3	0.76	3.12	<0.001	3
C18orf16	0.76	3.12	<0.001	0.79
FBXO17	0.76	2.22	<0.001	1.47

LGI4	0.76	2.22	<0.001	1.11
LOC402377	0.76	3.12	<0.001	1.21
C3orf36	0.76	3.12	<0.001	-0.79
BEGAIN	0.76	2.22	<0.001	0.56
HECW2	0.76	2.22	0.047	-0.2
KLRD1	0.76	3.12	<0.001	0.84
C12orf69	0.76	3.12	<0.001	2
SLITRK5	0.76	3.12	<0.001	3.92
CHRNA6	0.76	3.12	0.002	-0.6
ABCC4	0.76	2.22	<0.001	1.74
MYO7A	0.76	2.22	0.014	-0.32
ISM1	0.76	3.12	<0.001	1.67
CEACAM21	0.76	3.12	0.253	0.29
PGM5P2	0.76	2.22	<0.001	1.05
GPR174	0.76	3.12	<0.001	1.44
STX11	0.76	2.22	<0.001	1.49
FBXW10	0.76	3.12	<0.001	2.69
FAM81B	0.76	3.12	<0.001	1.28
TSPAN7	0.76	2.22	<0.001	1.09
SCML4	0.76	2.22	<0.001	1.39
JAKMIP1	0.76	2.22	<0.001	1.74
CPXM2	0.75	3.12	<0.001	1.46
HSPB6	0.75	2.22	<0.001	2.08
MYO1H	0.75	3.12	<0.001	1.71
NRG2	0.75	2.22	<0.001	1.16
TRPV6	0.75	3.12	<0.001	1.23
PSAT1	0.75	2.22	<0.001	0.55
BTBD11	0.75	3.12	<0.001	1.67
SGCD	0.75	2.22	<0.001	2.36
SCUBE2	0.75	2.22	<0.001	0.9
C20orf26	0.75	3.12	<0.001	2.06
MCART6	0.75	2.22	<0.001	0.65
RPS6KA6	0.75	2.22	<0.001	2.25
ANKRD20B	0.75	2.22	<0.001	1.94
QPRT	0.75	2.22	0.922	-0.07
FAM81A	0.75	2.22	0.03	0.28
CD300C	0.75	3.12	0.277	-0.06
GNMT	0.75	2.22	<0.001	1.25
BATF2	0.75	2.22	0.059	0.2
KIF1A	0.75	2.22	<0.001	2.52
TSNAXIP1	0.75	3.12	<0.001	2.13
LOC389493	0.75	3.12	<0.001	1.19
ZNF826	0.75	2.22	<0.001	1.48
IGF2BP3	0.75	2.22	<0.001	0.94
SUSD5	0.75	2.22	<0.001	0.97
SDF2L1	0.75	3.12	<0.001	0.85
SDPR	0.75	2.22	<0.001	1.61
ITPRIPL1	0.75	2.22	0.082	-0.22
SLC29A4	0.75	3.12	<0.001	2.18
CD96	0.75	2.22	<0.001	0.97
PCDHGA2	0.75	2.22	0.276	0.31
C5orf20	0.75	2.22	0.329	0.32
FCN3	0.75	2.22	<0.001	-1.52
EFHB	0.75	2.22	<0.001	2.14
RGL4	0.75	2.22	0.049	0.4

CCDC141	0.75	2.22	<0.001	1.6
ART4	0.75	2.22	<0.001	2.85
CNTFR	0.75	2.22	<0.001	3.19
LOC728264	0.75	2.22	<0.001	-0.49
ITGAL	0.75	2.22	0.006	0.64
SGCA	0.75	2.22	0.066	0.38
ISLR2	0.75	2.22	0.001	-0.44
LOC653786	0.75	2.22	<0.001	-0.99
GRAMD1B	0.75	2.22	<0.001	1.17
HRC	0.75	1.28	0.464	0.02
CCDC85A	0.75	2.22	<0.001	1.61
KLF2	0.75	2.22	<0.001	0.66
SIGLEC11	0.75	2.22	<0.001	1.53
COX7A1	0.75	1.28	0.068	0.43
ATP6V1G2	0.75	2.22	<0.001	1.02
CX3CR1	0.75	2.22	<0.001	-0.54
MYCBPAP	0.75	2.22	<0.001	0.84
UNC45B	0.75	2.22	<0.001	0.59
C9orf66	0.75	2.22	<0.001	1.43
SLC6A17	0.75	2.22	<0.001	0.95
C16orf89	0.75	2.22	<0.001	1.21
DERL3	0.75	2.22	0.838	-0.11
MYEOV	0.75	2.22	0.223	0.2
CMKLR1	0.75	2.22	<0.001	0.8
PLXNB3	0.75	2.22	0.193	-0.15
TPTE2P1	0.75	2.22	<0.001	3.36
LQK1	0.75	2.22	0.529	0.15
SLC17A7	0.75	1.28	<0.001	2.42
ABCA10	0.75	2.22	<0.001	2.21
DLX6	0.75	2.22	0.026	-0.34
GALNT14	0.74	2.22	0.02	-0.36
TLR7	0.74	2.22	0.062	-0.33
PURG	0.74	2.22	<0.001	0.83
RASGRP2	0.74	2.22	0.019	0.64
PCBP3	0.74	2.22	0.077	0.35
VNN2	0.74	2.22	0.204	0.46
GGTLC1	0.74	2.22	<0.001	0.62
ANK2	0.74	2.22	<0.001	3.06
ITGA8	0.74	1.28	<0.001	1.46
NCRNA00173	0.74	2.22	<0.001	1.07
PPM1L	0.74	2.22	<0.001	1.64
ABCG4	0.74	2.22	<0.001	-1.36
CD38	0.74	2.22	<0.001	1.73
C1QTNF9	0.74	2.22	<0.001	1
PRR15L	0.74	1.28	<0.001	1.33
TPPP	0.74	2.22	<0.001	2.48
CRABP1	0.74	2.22	<0.001	3.92
CD6	0.74	1.28	0.132	0.47
PRR18	0.74	2.22	<0.001	1.27
CHRNA3	0.74	2.22	0.001	0.65
TMC3	0.74	2.22	0.008	-0.54
ATP1B2	0.74	2.22	0.203	0.24
OLFM2	0.74	2.22	<0.001	1.4
OXCT2	0.74	2.22	<0.001	0.81
TRIM50	0.74	2.22	<0.001	1.93

LMOD1	0.74	2.22	<0.001	2.68
TRPM6	0.74	2.22	<0.001	1.16
GOLGA9P	0.74	2.22	<0.001	1.94
BCL11B	0.74	1.28	<0.001	1.71
SLC24A3	0.74	2.22	<0.001	2.02
FABP5	0.74	2.22	<0.001	0.68
GLIPR1L2	0.74	2.22	<0.001	1.13
SFTPC	0.74	2.22	<0.001	2.79
TCEAL2	0.74	2.22	<0.001	3.37
MX2	0.74	2.22	0.221	-0.14
CCDC48	0.74	1.28	0.001	0.48
DNAJC12	0.74	1.28	0.038	0.27
PKNOX2	0.74	2.22	<0.001	3.45
GPR20	0.74	2.22	0.307	0.17
PPP1R3C	0.74	2.22	0.836	-0.03
CYP7B1	0.74	1.28	<0.001	1.84
IL33	0.74	2.22	<0.001	1.55
EYA4	0.74	1.28	<0.001	1.98
PCDP1	0.74	2.22	<0.001	2.2
PIK3CG	0.74	2.22	0.137	0.42
FAM181A	0.74	1.28	<0.001	0.91
NMUR2	0.74	1.28	<0.001	1.61
SLC4A5	0.74	1.28	0.005	0.42
EDN2	0.74	2.22	0.084	-0.33
C13orf15	0.74	1.28	0.581	0.03
TUBA3D	0.74	1.28	<0.001	0.67
PLA2G4F	0.74	1.28	<0.001	2.03
UGT8	0.74	1.28	<0.001	2.03
GPR141	0.74	1.28	0.008	-0.59
OSBPL6	0.74	1.28	<0.001	1.46
SLC15A2	0.74	1.28	<0.001	0.77
FLJ35024	0.74	2.22	<0.001	2.06
NKG7	0.74	1.28	0.13	0.34
TSPAN18	0.74	1.28	0.283	-0.2
FLT1	0.74	1.28	0.028	-0.22
CORO2B	0.74	1.28	<0.001	1.63
PDCD1	0.73	1.28	<0.001	1.02
AOAH	0.73	1.28	<0.001	0.89
GPER	0.73	1.28	<0.001	0.83
PCSK1	0.73	1.28	<0.001	1.06
LHFPL1	0.73	2.22	<0.001	0.94
ENPP3	0.73	1.28	0.018	0.09
HOXA3	0.73	1.28	<0.001	0.61
SCUBE1	0.73	2.22	<0.001	0.59
RNF157	0.73	1.28	<0.001	0.97
SLC16A8	0.73	1.28	0.001	0.35
PROX1	0.73	1.28	<0.001	2.89
NEB	0.73	2.22	<0.001	3.06
FAM3D	0.73	1.28	<0.001	-0.7
LRRC4C	0.73	0.91	<0.001	2.37
TNNI3K	0.73	1.28	<0.001	0.95
PCDHB15	0.73	1.28	<0.001	0.62
RASSF6	0.73	2.22	<0.001	3.51
LOC100129726	0.73	1.28	<0.001	1.59
CARD16	0.73	1.28	0.189	-0.15

PAH	0.73	1.28	<0.001	1.39
HKDC1	0.73	1.28	<0.001	2.24
KLRK1	0.73	1.28	<0.001	1.31
GPR83	0.73	1.28	<0.001	2.33
EDA	0.73	1.28	0.003	0.73
FBXO15	0.73	2.22	<0.001	1.93
HAND2	0.73	1.28	<0.001	2.06
OTOS	0.73	2.22	<0.001	2.53
PRF1	0.73	1.28	<0.001	0.63
FAM129C	0.73	1.28	0.14	1.07
LOC115110	0.73	1.28	<0.001	-0.4
MPEG1	0.73	1.28	0.361	0.23
EFCAB5	0.73	1.28	<0.001	0.67
BLK	0.73	1.28	<0.001	2.34
ACSL6	0.73	1.28	<0.001	2.24
C10orf128	0.73	2.22	0.301	0.21
LOC342346	0.73	1.28	<0.001	1.74
DHH	0.73	2.22	<0.001	-0.49
CYP26B1	0.73	1.28	<0.001	1.63
ADCY1	0.73	1.28	<0.001	0.64
EXOC3L	0.73	0.91	<0.001	-0.37
CAPSL	0.73	1.28	<0.001	2.55
RFPL1S	0.73	1.28	<0.001	1.29
C1orf127	0.73	1.28	0.052	0.27
CLMN	0.73	1.28	<0.001	2.07
INHBC	0.73	1.28	<0.001	1.73
TMEM90B	0.73	1.28	<0.001	2.49
RAPGEF4	0.73	0.91	<0.001	-0.45
ZNF323	0.73	1.28	<0.001	0.74
WDR65	0.73	1.28	<0.001	1.76
BOC	0.73	0.91	<0.001	0.53
CDYL2	0.73	1.28	0.747	0.03
BST2	0.73	1.28	0.346	0.14
PLA2G3	0.73	1.28	0.001	0.76
HS3ST3B1	0.73	1.28	<0.001	0.89
ROPN1L	0.73	1.28	<0.001	1.21
COL4A6	0.73	1.28	<0.001	2.43
SUCNR1	0.73	1.28	<0.001	2.13
FOXF2	0.73	0.91	<0.001	1.04
LOC283174	0.73	1.28	<0.001	1.78
DLX1	0.73	0.91	<0.001	0.68
C9orf152	0.73	0.91	<0.001	1.34
PRIMA1	0.73	1.28	<0.001	2.94
DDIT4L	0.73	1.28	<0.001	1.62
CD163L1	0.72	0.91	<0.001	1.04
C10orf79	0.72	1.28	<0.001	1.96
COL9A3	0.72	1.28	<0.001	3.11
PDE11A	0.72	1.28	<0.001	2.63
C9orf43	0.72	1.28	<0.001	1.32
SYT6	0.72	1.28	0.003	-0.6
LRRN2	0.72	1.28	0.008	0.4
GP6	0.72	2.22	<0.001	0.88
DEPDC6	0.72	1.28	<0.001	2.11
UPF0639	0.72	1.28	<0.001	3.43
ADAM22	0.72	0.91	<0.001	2.87

PDE7B	0.72	0.91	0.353	0.2
SAMD3	0.72	1.28	0.021	0.56
ZNF521	0.72	0.91	<0.001	0.79
FMO2	0.72	0.91	<0.001	2.89
TOX	0.72	1.28	<0.001	1.97
AKR1C1	0.72	1.28	<0.001	2.73
FAM166B	0.72	1.28	0.121	0.23
TDH	0.72	1.28	<0.001	2.56
GZMK	0.72	1.28	<0.001	1.42
NOL4	0.72	1.28	<0.001	4.25
CDHR3	0.72	1.28	<0.001	3.09
SH2D6	0.72	1.28	<0.001	3.53
DOCK10	0.72	0.91	<0.001	-0.73
SSPO	0.72	1.28	<0.001	1.12
CECR1	0.72	0.91	0.164	0.38
VPREB3	0.72	1.28	<0.001	1.38
GCSH	0.72	1.28	<0.001	1.53
KCNIP3	0.72	1.28	<0.001	1.35
CCDC68	0.72	0.91	<0.001	0.82
GRRP1	0.72	0.91	0.258	0.13
AKR1E2	0.72	1.28	<0.001	2.38
FGF12	0.72	1.28	0.001	0.35
VAV3	0.72	0.91	0.45	-0.15
PRKCA	0.72	0.91	<0.001	1.33
PLVAP	0.72	0.91	0.002	-0.38
DUOXA2	0.72	0.91	0.12	0.22
GPR62	0.72	0.91	<0.001	0.96
DACH2	0.72	1.28	<0.001	-1.92
CD8A	0.72	1.28	<0.001	1.2
TXNDC6	0.72	1.28	<0.001	0.47
CACNA2D3	0.72	0.91	<0.001	1.56
C7orf58	0.72	0.91	<0.001	1.3
C12orf39	0.72	0.91	<0.001	3.44
EPB41L3	0.72	0.91	<0.001	0.94
FAM167A	0.72	1.28	<0.001	2.73
TMSB15A	0.72	1.28	<0.001	0.67
GPR34	0.72	0.91	0.3	-0.11
C11orf21	0.72	1.28	0.078	0.38
COL25A1	0.72	0.91	<0.001	2.07
TCF7L1	0.72	0.91	<0.001	1.67
HOXA2	0.72	0.38	<0.001	0.63
AMDHD1	0.72	1.28	<0.001	0.68
RTN1	0.71	0.91	<0.001	1.44
SH2D1B	0.71	1.28	0.028	0.4
SPARCL1	0.71	0.91	0.007	0.34
KCNK2	0.71	1.28	<0.001	3.14
FABP4	0.71	0.91	<0.001	3.26
SSTR1	0.71	1.28	0.001	0.5
AR	0.71	0.91	<0.001	2.41
TBX21	0.71	0.91	<0.001	0.87
KITLG	0.71	0.38	0.023	0.33
LRRC55	0.71	0.91	0.361	-0.1
CACNA2D2	0.71	0.91	0.853	0.01
TNFRSF11B	0.71	0.91	<0.001	2.82
ST6GALNAC1	0.71	1.28	0.644	-0.03

SLC5A5	0.71	1.28	<0.001	6.27
C9orf122	0.71	0.91	<0.001	1.17
PLB1	0.71	0.91	<0.001	1.16
SERHL2	0.71	0.91	<0.001	2.64
MYH11	0.71	0.91	<0.001	1.68
TNFSF8	0.71	0.91	0.004	0.7
RBP5	0.71	0.91	<0.001	1.05
RERG	0.71	0.38	0.059	0.28
FAM19A1	0.71	1.28	<0.001	1.04
SCG3	0.71	1.28	<0.001	2.6
WDR63	0.71	0.91	<0.001	0.63
PREX2	0.71	0.91	<0.001	2.13
ADH1B	0.71	0.91	<0.001	5.17
TSPAN33	0.71	0.91	<0.001	1.04
DIO2	0.71	0.38	<0.001	2.04
MT1M	0.71	0.91	<0.001	2.39
FAM162B	0.71	0.38	0.002	0.59
FGL2	0.71	0.91	<0.001	1.79
HS6ST3	0.71	0.91	<0.001	3.15
MAOB	0.71	0.91	<0.001	1.6
SCN4B	0.71	0.38	<0.001	0.95
RBM20	0.71	0.91	<0.001	1.18
C14orf50	0.71	0.91	<0.001	1.01
ASXL3	0.71	0.91	<0.001	3.79
KCNK17	0.71	0.38	0.001	0.72
SELP	0.71	0.91	0.001	-0.43
SLC25A34	0.71	0.91	<0.001	0.67
ART5	0.71	0.91	<0.001	2.63
C16orf74	0.71	0.91	<0.001	0.56
ABCG2	0.71	1.28	<0.001	-0.49
CPNE5	0.71	0.91	<0.001	-0.67
FAM155B	0.71	0.91	<0.001	1.32
GPC3	0.71	0.91	<0.001	4.46
C8orf84	0.71	0.91	<0.001	2.44
FAM26F	0.71	0.91	0.61	-0.08
BMX	0.71	0.91	<0.001	0.54
QRICH2	0.71	0.91	<0.001	1.32
TPO	0.70	0.91	<0.001	4.42
ANKRD29	0.70	0.38	<0.001	1.53
MEOX2	0.70	0.38	<0.001	1.65
KCNC1	0.70	0.91	<0.001	0.68
F10	0.70	0.91	<0.001	3.06
GFRA1	0.70	0.91	<0.001	3.55
AGMAT	0.70	0.38	<0.001	0.49
P2RY8	0.70	0.38	0.432	0.16
COL9A1	0.70	0.91	0.307	-0.19
PACSIN1	0.70	0.91	<0.001	-0.78
AKR1C2	0.70	0.91	<0.001	2.92
JPH4	0.70	0.91	0.14	-0.12
DHDPSL	0.70	0.38	<0.001	2.25
GPX3	0.70	0.91	<0.001	0.97
FOXJ1	0.70	0.91	<0.001	3.81
AQP4	0.70	0.38	<0.001	2.18
MYCN	0.70	0.38	<0.001	1.41
TDO2	0.70	0.91	<0.001	-1.57

DPY19L2P4	0.70	0.91	<0.001	2.16
BMP8A	0.70	0.91	<0.001	4.14
CD27	0.70	0.38	0.007	0.62
NPL	0.70	0.91	0.609	0.01
ATP2C2	0.70	0.38	<0.001	3.74
AVPR2	0.70	0.91	0.063	0.29
TMEM47	0.70	0.38	<0.001	1.08
PYGM	0.70	0.91	<0.001	2.15
COCH	0.70	0.38	<0.001	0.88
WFDC1	0.70	0.38	0.019	0.32
AVPR1A	0.70	0.38	<0.001	3.29
LOC151174	0.70	0.91	0.004	-0.42
PTPRQ	0.70	0.38	<0.001	3.87
DOC2A	0.70	0.38	0.112	-0.22
MAPK4	0.70	0.91	<0.001	4.31
SMAD5OS	0.70	0.38	<0.001	0.95
MT1H	0.70	0.91	<0.001	4.06
LOC84856	0.70	0.38	<0.001	0.96
C3orf55	0.70	0.38	<0.001	2.2
CLEC3B	0.70	0.38	0.177	0.19
DLEU7	0.70	0.38	<0.001	0.76
EXOC3L2	0.70	0.38	0.001	-0.43
PLCH1	0.70	0.38	<0.001	3.05
MMRN1	0.69	0.91	<0.001	3.88
FAM13C	0.69	0.38	<0.001	1.15
FGD2	0.69	0.91	<0.001	1.4
RANBP3L	0.69	0.38	<0.001	2.79
EMID1	0.69	0.38	<0.001	0.94
LRRC70	0.69	0.38	0.001	0.42
FLT4	0.69	0.25	0.003	0.4
PID1	0.69	0.38	<0.001	3.06
ELMO1	0.69	0.38	<0.001	2.39
LRMP	0.69	0.38	0.209	0.43
ANO3	0.69	0.38	<0.001	0.97
C6orf165	0.69	0.38	<0.001	1.68
LRRC2	0.69	0.38	<0.001	1.45
ATRNL1	0.69	0.38	<0.001	1.22
GPR37L1	0.69	0.91	<0.001	1.98
ABCC8	0.69	0.38	<0.001	2.37
FGFBP2	0.69	0.38	<0.001	1.22
DPT	0.69	0.38	<0.001	5.53
FREM2	0.69	0.38	<0.001	1.48
SUSD2	0.69	0.38	<0.001	-0.42
FRZB	0.69	0.00	0.344	0.19
CASP12	0.69	0.38	<0.001	1.03
GZMM	0.69	0.38	<0.001	0.94
KIT	0.69	0.38	<0.001	3.22
LOC100128164	0.69	0.38	<0.001	1.35
PDE1B	0.69	0.38	0.339	-0.09
EFHC2	0.69	0.38	<0.001	1.7
SLC16A2	0.69	0.38	<0.001	1.62
LOC400759	0.69	0.25	<0.001	1.02
SHANK2	0.69	0.25	<0.001	1.95
ZNF541	0.69	0.91	<0.001	1.31
GRB14	0.69	0.38	<0.001	1.09

CACNB2	0.69	0.25	0.484	-0.02
NCF1B	0.69	0.38	0.11	0.48
XK	0.69	0.38	<0.001	0.82
INPP5J	0.69	0.38	<0.001	0.73
IPCEF1	0.69	0.38	<0.001	4.09
KATNAL2	0.69	0.38	<0.001	1.82
SLC1A2	0.69	0.38	<0.001	1.65
LOC400043	0.69	0.25	0.033	0.27
STXBP5L	0.69	0.38	<0.001	4.37
USHBP1	0.69	0.38	0.028	-0.23
EFEMP1	0.69	0.38	<0.001	2.26
COLEC10	0.69	0.38	<0.001	2.54
RIMKLA	0.69	0.25	<0.001	1.81
MYH3	0.69	0.25	<0.001	1.32
SELV	0.69	0.38	<0.001	3.45
MS4A1	0.69	0.38	0.017	1.3
TRPV4	0.69	0.25	0.014	-0.22
RGNEF	0.69	0.38	<0.001	1.79
MASP1	0.69	0.38	<0.001	3.61
EBF4	0.69	0.25	<0.001	1.64
SLC13A4	0.69	0.38	<0.001	1.12
SULT1C4	0.69	0.25	<0.001	1.58
SLC5A4	0.68	0.38	0.997	0.01
ARHGEF37	0.68	0.25	<0.001	1.15
PRKG1	0.68	0.25	<0.001	2
LILRB5	0.68	0.25	0.421	0.11
DPY19L2	0.68	0.38	<0.001	1.76
EOMES	0.68	0.38	<0.001	1.02
LOC201651	0.68	0.25	<0.001	3.95
ME1	0.68	0.38	<0.001	1
RGS9	0.68	0.25	<0.001	1.37
RADIL	0.68	0.25	<0.001	3.05
KCNA3	0.68	0.25	<0.001	1.05
LIPG	0.68	0.25	<0.001	1.23
SLC26A7	0.68	0.25	<0.001	3.43
CDCA7	0.68	0.38	<0.001	-0.68
C15orf51	0.68	0.38	<0.001	1.25
FHL5	0.68	0.25	0.003	0.58
C13orf36	0.68	0.25	<0.001	4.04
C19orf35	0.68	0.38	0.337	-0.15
SP6	0.68	0.25	0.465	0.1
MT1L	0.68	0.38	<0.001	1.11
ASCL2	0.68	0.38	<0.001	0.81
CWH43	0.68	0.25	<0.001	4.35
LGI3	0.68	0.25	<0.001	2.18
ARHGAP28	0.68	0.25	<0.001	1.28
SCN3B	0.68	0.25	<0.001	1.41
REM1	0.68	0.00	0.295	-0.11
TMEM88	0.68	0.25	0.705	0.09
MT1F	0.68	0.38	<0.001	2.68
GPR98	0.68	0.25	<0.001	3.62
ROBO4	0.68	0.25	0.085	-0.18
MOSC1	0.68	0.25	<0.001	1.09
CXCL12	0.67	0.00	<0.001	1.43
PRKCB	0.67	0.00	0.568	0.01

CETP	0.67	0.25	0.007	0.62
LOC100272216	0.67	0.00	<0.001	1.65
CD36	0.67	0.25	<0.001	1.53
SYN3	0.67	0.25	<0.001	0.62
LAMC3	0.67	0.25	<0.001	0.96
APBA2	0.67	0.25	<0.001	1.09
TAL1	0.67	0.00	<0.001	0.72
MAB21L2	0.67	0.25	<0.001	1.45
SLCO1C1	0.67	0.25	<0.001	-0.94
PFKFB1	0.67	0.38	<0.001	2.22
PLA1A	0.67	0.25	<0.001	0.82
LOC442421	0.67	0.25	<0.001	1.31
LRRTM1	0.67	0.25	<0.001	1.6
MYOM1	0.67	0.00	<0.001	0.92
MATN2	0.67	0.25	<0.001	2.79
ANKK1	0.67	0.00	<0.001	2.22
MLC1	0.67	0.25	0.411	0.14
C9orf24	0.67	0.25	<0.001	2.11
CA4	0.67	0.00	<0.001	3.01
KHDRBS2	0.67	0.25	<0.001	3.28
CR2	0.67	0.25	<0.001	2.1
CLEC9A	0.67	0.25	0.341	0.3
RTN4RL1	0.67	0.25	0.373	0.22
CCL14	0.67	0.00	<0.001	1.33
QRFPR	0.67	0.00	<0.001	-1.56
LOC100134868	0.67	0.25	<0.001	1.5
ARHGAP24	0.67	0.25	<0.001	2.27
TRPC6	0.67	0.00	<0.001	0.87
RGS7BP	0.67	0.25	<0.001	1.37
PAPSS2	0.66	0.38	<0.001	2.94
IGDCC4	0.66	0.00	0.14	0.13
IP6K3	0.66	0.25	<0.001	2.69
CDHR1	0.66	0.25	<0.001	1.25
LRP1B	0.66	0.25	<0.001	5.87
FRMPD1	0.66	0.00	<0.001	1.94
CDON	0.66	0.25	<0.001	2.14
OCA2	0.66	0.25	<0.001	4.38
TMEM38A	0.66	0.25	<0.001	0.85
MYCL1	0.66	0.00	<0.001	1.07
ANK1	0.66	0.25	<0.001	0.77
RSPH4A	0.66	0.00	<0.001	1.63
LRP2	0.66	0.00	<0.001	2.17
CUX2	0.66	0.00	<0.001	5.2
KCNJ10	0.66	0.00	0.002	0.53
TMEM171	0.66	0.25	<0.001	2.77
SLC14A2	0.66	0.25	<0.001	1.24
SLC26A4	0.66	0.00	<0.001	3.24
HAP1	0.66	0.25	<0.001	2.99
DIRAS2	0.66	0.00	<0.001	2.98
ITPR1	0.66	0.00	<0.001	2.59
B3GALT2	0.66	0.00	<0.001	3.23
PTCH2	0.66	0.00	<0.001	1.68
GPR17	0.66	0.25	<0.001	0.72
C2orf73	0.66	0.25	<0.001	1.85
RASSF9	0.66	0.00	<0.001	3

CABP1	0.66	0.00	<0.001	-0.57
DPP6	0.66	0.00	<0.001	5.5
GP5	0.66	0.00	<0.001	2.07
SORCS1	0.66	0.00	<0.001	1.48
ANGPT1	0.66	0.00	0.006	0.41
NFASC	0.66	0.00	<0.001	0.91
CACNB4	0.66	0.00	<0.001	1.28
KDR	0.66	0.00	0.501	-0.03
ADRB1	0.66	0.00	0.007	0.32
CNTN5	0.66	0.00	<0.001	4.56
FMO1	0.65	0.00	<0.001	2.34
C14orf64	0.65	0.00	<0.001	1.99
MIOX	0.65	0.25	<0.001	2.69
SEMA3D	0.65	0.00	<0.001	5.08
PTGDR	0.65	0.00	<0.001	1.04
ADCY5	0.65	0.00	<0.001	1.06
CXXC4	0.65	0.00	<0.001	1.82
L3MBTL4	0.65	0.00	<0.001	1.64
NMUR1	0.65	0.00	<0.001	1.1
BCO2	0.65	0.00	<0.001	1.77
ZNF366	0.65	0.00	0.913	0.1
SCN3A	0.65	0.00	<0.001	2.51
RYR2	0.65	0.00	<0.001	4.57
PI16	0.65	0.00	<0.001	4.22
PRTG	0.65	0.00	<0.001	1.86
FHL1	0.65	0.00	<0.001	2.42
LOC286002	0.65	0.00	<0.001	3.1
FAM189A1	0.65	0.00	<0.001	2.96
SDK2	0.65	0.00	<0.001	2.03
RXFP1	0.65	0.00	<0.001	2.56
SGK1	0.65	0.00	<0.001	0.98
IL22RA1	0.65	0.00	<0.001	0.69
DIO1	0.65	0.00	<0.001	4.33
ZMAT4	0.65	0.00	<0.001	4.98
CETN4P	0.65	0.00	<0.001	0.95
ANGPTL1	0.65	0.00	<0.001	2.32
LOC90246	0.65	0.00	0.083	0.23
AKAP3	0.64	0.00	<0.001	1.52
RELN	0.64	0.00	<0.001	5.54
ST3GAL6	0.64	0.00	0.461	-0.06
AOX1	0.64	0.00	<0.001	4.48
AGTR1	0.64	0.00	<0.001	2.41
TFCP2L1	0.64	0.00	<0.001	3.84
USP44	0.64	0.00	<0.001	1.41
HGD	0.64	0.00	<0.001	3.25
SLC4A4	0.64	0.00	<0.001	3.45
KCNAB1	0.64	0.00	<0.001	1.85
LIFR	0.64	0.00	<0.001	2.46
FAM163A	0.64	0.00	<0.001	3.64
FAM124A	0.64	0.00	<0.001	2.17
IGFBPL1	0.64	0.00	<0.001	4.16
ACE	0.64	0.00	0.001	0.38
AXIN2	0.64	0.00	<0.001	1.26
EMCN	0.64	0.00	<0.001	0.59
CNTN2	0.64	0.00	<0.001	1.01

ANO2	0.63	0.00	0.564	0.05
MT1G	0.63	0.00	<0.001	3.83
DNASE1L3	0.63	0.00	<0.001	1.18
GBP4	0.63	0.00	0.153	0.14
IRS1	0.63	0.00	<0.001	1.71
MYRIP	0.63	0.00	<0.001	0.64
RAG1	0.63	0.00	<0.001	2.19
ESRRG	0.63	0.00	<0.001	1.7
NWD1	0.63	0.00	<0.001	3.94
ALDH1A1	0.63	0.00	<0.001	1.68
PKHD1L1	0.63	0.00	<0.001	5.54
GLDC	0.63	0.00	<0.001	3.97
GDF10	0.63	0.00	<0.001	4.27
CLEC4E	0.63	0.00	<0.001	1.36
SLC5A8	0.63	0.00	<0.001	4.61
REEP1	0.63	0.00	<0.001	0.94
COLEC11	0.62	0.00	<0.001	1.16
SIDT1	0.62	0.00	0.214	0.32
PAMR1	0.62	0.00	<0.001	0.69
GPR126	0.62	0.00	0.333	0.14
DLG2	0.62	0.00	<0.001	3.54
ECEL1	0.62	0.00	0.045	-0.49
FCGBP	0.62	0.00	<0.001	1.9
VWF	0.62	0.00	0.001	-0.43
GPM6A	0.62	0.00	<0.001	3.52
RCAN2	0.62	0.00	<0.001	1.05
KIAA1324	0.62	0.00	<0.001	3.54
GRIK4	0.62	0.00	<0.001	3.05
SHISA2	0.62	0.00	0.17	0.25
KCNJ13	0.62	0.00	<0.001	2.83
CLCNKA	0.62	0.00	<0.001	2.89
SAMD5	0.62	0.00	<0.001	2.1
RUNX1T1	0.62	0.00	<0.001	1.97
TM4SF18	0.62	0.00	0.435	-0.06
SORBS2	0.62	0.00	<0.001	2.28
SYNE1	0.62	0.00	<0.001	2.07
C3orf32	0.62	0.00	0.001	0.7
FZD9	0.62	0.00	<0.001	1.12
VIPR1	0.62	0.00	<0.001	1.28
SLC14A1	0.62	0.00	<0.001	1.18
CCL21	0.61	0.00	<0.001	6.4
LRRC4	0.61	0.00	<0.001	-1.4
NOSTRIN	0.61	0.00	0.31	-0.12
SYNM	0.61	0.00	<0.001	1.2
RASL11A	0.61	0.00	<0.001	0.89
C1QTNF9B	0.61	0.00	0.625	-0.04
NOX5	0.61	0.00	0.005	-0.91
PCSK6	0.61	0.00	<0.001	2.25
GVIN1	0.61	0.00	<0.001	1.31
GNA14	0.61	0.00	<0.001	1.91
PEAR1	0.61	0.00	<0.001	0.87
DUSP26	0.61	0.00	<0.001	0.98
LOC100130238	0.61	0.00	<0.001	3.41
CDH16	0.61	0.00	<0.001	5.47
GRIN2C	0.61	0.00	<0.001	3.52

EBF3	0.60	0.00	<0.001	0.7
KY	0.60	0.00	<0.001	2.37
MPPED2	0.60	0.00	<0.001	3.55
C8orf80	0.60	0.00	<0.001	3.74
RERGL	0.60	0.00	<0.001	3.09
FAM124B	0.60	0.00	<0.001	-0.76
PLA2R1	0.60	0.00	<0.001	3.56
SPINK5	0.60	0.00	<0.001	1.32
GABRB3	0.60	0.00	<0.001	2.34
FLRT1	0.59	0.00	<0.001	3.24
GCK	0.59	0.00	<0.001	1.72
SLC10A4	0.59	0.00	0.002	-0.55
CLCNKB	0.59	0.00	<0.001	2.86
CGNL1	0.59	0.00	<0.001	1.29
SYNGR1	0.59	0.00	<0.001	1.07
IQGAP2	0.59	0.00	<0.001	1.95
GRIP2	0.59	0.00	0.017	0.46
MAP2K6	0.59	0.00	<0.001	0.94
CD300LG	0.59	0.00	<0.001	2.19
LOC145820	0.58	0.00	<0.001	1.05
IGDCC3	0.58	0.00	0.005	0.77
TFF3	0.58	0.00	<0.001	6.06
MRO	0.58	0.00	<0.001	4.32
AQP7	0.58	0.00	<0.001	1.06
SOD3	0.57	0.00	<0.001	2.3
PGM5	0.57	0.00	<0.001	1.65
LRRC4B	0.56	0.00	0.218	-0.17
KIF19	0.56	0.00	<0.001	3.57
LTF	0.56	0.00	<0.001	3.02
FAM65C	0.56	0.00	<0.001	1.65
TDGF1	0.55	0.00	<0.001	2.71
WSCD2	0.55	0.00	<0.001	5.3
ABCB1	0.55	0.00	0.136	0.25
PTPRR	0.54	0.00	<0.001	-0.8
SYT15	0.48	0.00	<0.001	0.77

Supplementary table 3: Differential expression of prognostic genes in normal tissue, extreme good prognosis cancers and extreme poor prognosis cancers

Gene	Mean expression normal adjacent tissue	Mean expression extreme good prognosis (cancer)	Mean expression extreme poor prognosis (cancer)	Correlation coefficient* (direction of differential expression from normal to good prognosis to poor prognosis)	P-value** (Differential expression between cancer and normal)	Q-value (FDR) (Differential expression between cancer and normal)	Direction (In extreme prognosis relative to good prognosis)
AREG	3.75	3.57	5.12	0.3	<0.001	<0.001	Upregulated
NR1D1	7.92	9.34	10.43	0.68	<0.001	<0.001	Upregulated
FSTL3	8.45	9.24	10.13	0.61	<0.001	<0.001	Upregulated
IQGAP3	2.93	4.81	5.88	0.68	<0.001	<0.001	Upregulated
HSN2	2.67	2.17	2.88	0.1	0.595326475	0.595326475	Upregulated
SNX22	11.03	11.48	12.39	0.51	<0.001	<0.001	Upregulated
LIMS3	3.5	3.13	4.5	0.29	<0.001	<0.001	Upregulated
CELF4	3.19	5.32	6.51	0.74	<0.001	<0.001	Upregulated
SYT15	6.55	6.41	5.28	-0.53	<0.001	<0.001	Downregulated
LRRC4B	4.57	5.33	4.31	-0.15	0.217677594	0.219693128	Downregulated
LRRC4	3.93	5.87	4.97	0.32	<0.001	<0.001	Downregulated
FZD9	2.7	2.13	0.97	-0.52	<0.001	<0.001	Downregulated
SOD3	11.79	10.41	9.02	-0.71	<0.001	<0.001	Downregulated
ABCB1	6.78	7.08	6.08	-0.31	0.136357968	0.140217155	Downregulated
FAM65C	6.24	5.43	4.06	-0.6	<0.001	<0.001	Downregulated
LTF	9.41	7.53	5.77	-0.7	<0.001	<0.001	Downregulated
WSCD2	10.95	7.69	4.72	-0.73	<0.001	<0.001	Downregulated
TDGF1	4.76	3.07	1.48	-0.73	<0.001	<0.001	Downregulated
MRO	8.75	5.78	3.63	-0.73	<0.001	<0.001	Downregulated
PGM5	8.44	7.38	6.38	-0.68	<0.001	<0.001	Downregulated
RASL11A	6.74	6.5	5.63	-0.49	<0.001	<0.001	Downregulated
SPINK5	5.1	4.88	3.19	-0.39	<0.001	<0.001	Downregulated
TFF3	14.03	9.64	7.12	-0.76	<0.001	<0.001	Downregulated
SYNM	9.4	8.64	7.86	-0.67	<0.001	<0.001	Downregulated
PAMR1	6.93	6.79	5.97	-0.45	<0.001	<0.001	Downregulated
IGDCC3	5.38	5.7	4.05	-0.33	0.004768615	0.005046398	Downregulated
GRIP2	2.5	2.82	1.66	-0.3	0.016992077	0.017809003	Downregulated
PEAR1	8.09	7.72	6.93	-0.55	<0.001	<0.001	Downregulated

RERGL	5.72	3.78	2.18	-0.66	<0.001	<0.001	Downregulated
GABRB3	9.04	7.62	5.91	-0.57	<0.001	<0.001	Downregulated
GRIK4	7.04	5.15	3.47	-0.65	<0.001	<0.001	Downregulated
CGNL1	11.88	11.05	10.09	-0.59	<0.001	<0.001	Downregulated
FLRT1	7.93	5.84	4.06	-0.66	<0.001	<0.001	Downregulated
MPPED2	10.25	7.74	6.03	-0.72	<0.001	<0.001	Downregulated
KIF19	7	4.66	2.71	-0.71	<0.001	<0.001	Downregulated
GNA14	9.18	7.93	6.81	-0.66	<0.001	<0.001	Downregulated
IQGAP2	11.02	9.73	8.51	-0.65	<0.001	<0.001	Downregulated
GCK	4.07	3.27	1.8	-0.56	<0.001	<0.001	Downregulated
MAP2K6	5.77	5.35	4.53	-0.52	<0.001	<0.001	Downregulated
PCSK6	6.28	4.81	3.82	-0.71	<0.001	<0.001	Downregulated
REEP1	5.99	5.71	4.71	-0.45	<0.001	<0.001	Downregulated
GRIN2C	10.25	8.07	6.16	-0.65	<0.001	<0.001	Downregulated
FAM124B	4.12	5.43	4.67	0.19	<0.001	<0.001	Downregulated
NOX5	2.64	4.56	2.76	-0.01	0.004717782	0.005041551	Downregulated
SDK2	7.86	6.63	5.54	-0.64	<0.001	<0.001	Downregulated
CD300LG	4.74	3.65	2.17	-0.56	<0.001	<0.001	Downregulated
SORBS2	13.23	11.49	10.57	-0.73	<0.001	<0.001	Downregulated
SYNGR1	8.91	8.38	7.57	-0.57	<0.001	<0.001	Downregulated
RCAN2	9.52	8.96	8.14	-0.58	<0.001	<0.001	Downregulated
ACE	9.64	9.63	8.94	-0.36	<0.001	0.001016059	Downregulated
RUNX1T1	5.96	4.66	3.58	-0.64	<0.001	<0.001	Downregulated
SYNE1	12.55	11	10.19	-0.73	<0.001	<0.001	Downregulated
DLG2	9.93	7.28	6.06	-0.76	<0.001	<0.001	Downregulated
CDH16	11.18	7.25	4.97	-0.73	<0.001	<0.001	Downregulated
PLA2R1	11.47	8.69	7.54	-0.79	<0.001	<0.001	Downregulated
ECEL1	3.83	5.36	4.07	0.03	0.044716212	0.046419687	Downregulated
AXIN2	9.39	8.57	7.89	-0.64	<0.001	<0.001	Downregulated
C8orf80	7.33	4.9	3.13	-0.69	<0.001	<0.001	Downregulated
VWF	11.55	12.44	11.7	0.03	<0.001	<0.001	Downregulated
IRS1	11.84	10.54	9.82	-0.69	<0.001	<0.001	Downregulated
KIAA1324	9.79	7.41	5.8	-0.67	<0.001	<0.001	Downregulated
RELN	8.06	4.04	2.33	-0.75	<0.001	<0.001	Downregulated
CCL21	11.81	7.61	4.76	-0.71	<0.001	<0.001	Downregulated

C14orf64	5.99	4.58	3.68	-0.65	<0.001	<0.001	Downregulated
PI16	9.1	6.3	4.54	-0.67	<0.001	<0.001	Downregulated
CXCL12	11	10.3	9.45	-0.54	<0.001	<0.001	Downregulated
CNTN2	2.5	2.3	1.19	-0.41	<0.001	<0.001	Downregulated
FAM124A	6.2	4.68	3.77	-0.69	<0.001	<0.001	Downregulated
NWD1	7.44	4.58	2.86	-0.7	<0.001	<0.001	Downregulated
C13orf36	7.7	4.96	3.16	-0.66	<0.001	<0.001	Downregulated
ZMAT4	8.87	5.37	3.13	-0.67	<0.001	<0.001	Downregulated
HGD	10.33	7.94	6.46	-0.67	<0.001	<0.001	Downregulated
SLC5A8	10.48	7.18	4.98	-0.66	<0.001	<0.001	Downregulated
RYR2	9.87	6.25	4.87	-0.77	<0.001	<0.001	Downregulated
LAMC3	5.23	5.05	3.8	-0.35	<0.001	<0.001	Downregulated
OC10027221	4.66	3.66	2.8	-0.54	<0.001	<0.001	Downregulated
DUSP26	3.67	3.39	2.25	-0.4	<0.001	<0.001	Downregulated
LOC442421	4.39	3.54	2.83	-0.57	<0.001	<0.001	Downregulated
FAM163A	5.69	3.1	1.72	-0.7	<0.001	<0.001	Downregulated
SAMD5	7.39	6.01	4.89	-0.62	<0.001	<0.001	Downregulated
RAG1	6.93	5.37	4.33	-0.66	<0.001	<0.001	Downregulated
MYCL1	7.86	7.28	6.58	-0.5	<0.001	<0.001	Downregulated
PRTG	9.98	8.63	7.95	-0.67	<0.001	<0.001	Downregulated
PKHD1L1	12.35	8.38	5.98	-0.67	<0.001	<0.001	Downregulated
TFCP2L1	11.98	8.96	7.67	-0.74	<0.001	<0.001	Downregulated
LIFR	11.57	9.71	8.85	-0.71	<0.001	<0.001	Downregulated
DPP6	10.52	6.82	4.66	-0.69	<0.001	<0.001	Downregulated
GPM6A	9.1	6.37	5.16	-0.73	<0.001	<0.001	Downregulated
SLC4A4	11.34	8.68	7.32	-0.7	<0.001	<0.001	Downregulated
DIO1	12.17	9.32	7.12	-0.59	<0.001	<0.001	Downregulated
IGFBPL1	7.48	4.17	2.78	-0.75	<0.001	<0.001	Downregulated
ADCY5	3.8	3.49	2.41	-0.42	<0.001	<0.001	Downregulated
GVIN1	6.9	6.14	5.29	-0.49	<0.001	<0.001	Downregulated
SLC14A2	4.21	4.11	2.44	-0.31	<0.001	<0.001	Downregulated
HAP1	7.26	4.96	3.9	-0.72	<0.001	<0.001	Downregulated
CWH43	9.07	5.91	4.21	-0.68	<0.001	<0.001	Downregulated
RASSF9	6.74	4.3	3.48	-0.78	<0.001	<0.001	Downregulated
ARHGEF37	7.33	6.58	5.89	-0.58	<0.001	<0.001	Downregulated

SIDT1	5	5.19	4.38	-0.21	0.213856028	0.217853337	Downregulated
APBA2	7.23	6.74	5.85	-0.48	<0.001	<0.001	Downregulated
.OC10013486	3.04	1.97	1.22	-0.58	<0.001	<0.001	Downregulated
SEMA3D	10.97	7.23	5.15	-0.69	<0.001	<0.001	Downregulated
ZNF366	5.08	5.45	4.61	-0.19	0.912813957	0.912813957	Downregulated
CACNB4	6.48	5.78	4.84	-0.5	<0.001	<0.001	Downregulated
GDF10	7.7	4.87	3.01	-0.69	<0.001	<0.001	Downregulated
GP5	4.15	2.75	1.89	-0.64	<0.001	<0.001	Downregulated
ATP2C2	9.29	6.39	5.23	-0.74	<0.001	<0.001	Downregulated
AOX1	9.2	5.9	4.23	-0.72	<0.001	<0.001	Downregulated
PRKG1	7.21	5.75	4.74	-0.6	<0.001	<0.001	Downregulated
CXXC4	4.44	3.19	2.29	-0.57	<0.001	<0.001	Downregulated
RGS7BP	2.72	2.03	1.15	-0.45	<0.001	<0.001	Downregulated
LOC84856	2.38	1.96	1.19	-0.42	<0.001	<0.001	Downregulated
CUX2	7.91	4.04	2.09	-0.66	<0.001	<0.001	Downregulated
ITPR1	12.18	10.21	9.33	-0.71	<0.001	<0.001	Downregulated
GFRA1	7.9	5.3	3.97	-0.67	<0.001	<0.001	Downregulated
KIT	11.35	8.81	7.77	-0.71	<0.001	<0.001	Downregulated
EFEMP1	12.28	10.52	9.73	-0.69	<0.001	<0.001	Downregulated

correlation coefficient

gene expression between cancer and normal adjacent tissue overall

Supplementary Table 4. Genes consistently upregulated or downregulated (1) in extremem poor prognosis patients in leave-one-out cross validation

Genes upregulated in extreme poor prognosis patients relative to extreme good prognosis patients

Gene	Left out group (patients associated with specified poor prognositic clinical factor)							
	Adjuvant drug therapy	IMRT*	Persistent disease**	With tumor***	Died of PTC	Metastases present	New tumor event****	Sum*****
NR1D1	1	1	0	0	1	0	1	4
AREG	1	1	0	0	0	0	0	2
FSTL3	1	1	0	0	0	0	0	2
IQGAP3	1	1	0	0	0	0	0	2
SNX22	0	1	0	0	1	0	0	2
LIMS3	0	1	0	0	1	0	0	2
CELF4	0	1	0	0	1	0	0	2
HSN2	1	0	0	0	0	0	0	1

Genes downregulated in poor prognosis patients relative to extremem good prognosis patients

Gene	Left out group (patients associated with specified poor prognositic clinical factor)							
	Adjuvant drug therapy	IMRT**	Persistent disease**	With tumor***	Died of PTC	Metastases present	New tumor event****	Sum*****
SYT15	1	1	1	1	1	1	1	7
LRRC4B	1	1	1	1	1	1	1	7
LRRC4	1	1	1	1	1	1	1	7
FZD9	1	1	1	1	1	1	1	7
SOD3	1	1	1	1	1	1	1	7
ABCB1	1	1	1	1	1	1	1	7
FAM65C	1	1	1	1	1	1	1	7
LTF	1	1	1	1	1	1	1	7
WSCD2	1	1	1	1	1	1	1	7
TDGF1	1	1	1	1	1	1	1	7

MRO	1	1	1	1	1	1	1	7
PGM5	1	1	1	1	1	1	1	7
RASL11A	1	1	1	1	1	1	1	7
SPINK5	1	1	1	1	1	1	1	7
TFF3	1	1	1	1	1	1	1	7
SYNM	1	1	1	1	1	1	1	7
PAMR1	1	1	1	1	1	1	1	7
IGDCC3	1	1	1	1	1	1	1	7
GRIP2	1	1	1	1	1	1	1	7
PEAR1	1	1	1	1	1	1	1	7
RERGL	1	1	1	1	1	1	1	7
GABRB3	1	1	1	1	1	1	1	7
GRIK4	1	1	1	1	1	1	1	7
CGNL1	1	1	1	1	1	1	1	7
FLRT1	1	1	1	1	1	1	1	7
MPPED2	1	1	1	1	1	1	1	7
KIF19	1	1	1	1	1	1	1	7
GNA14	1	1	1	1	1	1	1	7
IQGAP2	1	1	1	1	1	1	1	7
GCK	1	1	1	1	1	1	1	7
MAP2K6	1	1	1	1	1	1	1	7
PCSK6	1	1	1	1	1	1	1	7
REEP1	1	1	1	1	1	1	1	7
GRIN2C	1	1	1	1	1	1	1	7
FAM124B	1	1	1	1	1	1	1	7
NOX5	1	1	1	1	1	1	1	7
SDK2	1	1	1	1	1	1	1	7
CD300LG	1	1	1	1	1	1	1	7
SORBS2	1	1	1	1	1	1	1	7
SYNGR1	1	1	1	1	1	1	1	7

RCAN2	1	1	1	1	1	1	1	7
ACE	1	1	1	1	1	1	1	7
RUNX1T1	1	1	1	1	1	1	1	7
SYNE1	1	1	1	1	1	1	1	7
DLG2	1	1	1	1	1	1	1	7
CDH16	1	1	1	1	1	1	1	7
PLA2R1	1	1	1	1	1	1	1	7
ECEL1	1	1	1	1	1	1	1	7
AXIN2	1	1	1	1	1	1	1	7
C8orf80	1	1	1	1	1	1	1	7
VWF	1	1	1	1	1	1	1	7
IRS1	1	1	1	1	1	1	1	7
KIAA1324	1	1	1	1	1	1	1	7
RELN	1	1	1	1	1	1	1	7
CCL21	1	1	1	1	1	1	1	7
C14orf64	1	1	1	1	1	1	1	7
PI16	1	1	1	1	1	1	1	7
CXCL12	1	1	1	1	1	1	1	7
CNTN2	1	1	1	1	1	1	1	7
FAM124A	1	1	1	1	1	1	1	7
NWD1	1	1	1	1	1	1	1	7
C13orf36	1	1	1	1	1	1	1	7
ZMAT4	1	1	1	1	1	1	1	7
HGD	1	1	1	1	1	1	1	7
SLC5A8	1	1	1	1	1	1	1	7
RYR2	1	1	1	1	1	1	1	7
LAMC3	1	1	1	1	1	1	1	7
LOC1002722	1	1	1	1	1	1	1	7
DUSP26	1	1	1	1	1	1	1	7
LOC442421	1	1	1	1	1	1	1	7

FAM163A	1	1	1	1	1	1	1	7
SAMD5	1	1	1	1	1	1	1	7
RAG1	1	1	1	1	1	1	1	7
MYCL1	1	1	1	1	1	1	1	7
PRTG	1	1	1	1	1	1	1	7
PKHD1L1	1	1	1	1	1	1	1	7
TFCP2L1	1	1	1	1	1	1	1	7
LIFR	1	1	1	1	1	1	1	7
DPP6	1	1	1	1	1	1	1	7
GPM6A	1	1	1	1	1	1	1	7
SLC4A4	1	1	1	1	1	1	1	7
DIO1	1	1	1	1	1	1	1	7
IGFBPL1	1	1	1	1	1	1	1	7
ADCY5	1	1	1	1	1	1	1	7
GVIN1	1	1	1	1	1	1	1	7
SLC14A2	1	1	1	1	1	1	1	7
HAP1	1	1	1	1	1	1	1	7
CWH43	1	1	1	1	1	1	1	7
RASSF9	1	1	1	1	1	1	1	7
ARHGEF37	1	1	1	1	1	1	1	7
SIDT1	1	1	1	1	1	1	1	7
APBA2	1	1	1	1	1	1	1	7
LOC1001348	1	1	1	1	1	1	1	7
SEMA3D	1	1	1	1	1	1	1	7
ZNF366	1	1	1	1	1	1	1	7
CACNB4	1	1	1	1	1	1	1	7
GDF10	1	1	1	1	1	1	1	7
GP5	1	1	1	1	1	1	1	7
ATP2C2	1	1	1	1	1	1	1	7
AOX1	1	1	1	1	1	1	1	7

PRKG1	1	1	1	1	1	1	1	7
CXXC4	1	1	1	1	1	1	1	7
RGS7BP	1	1	1	1	1	1	1	7
LOC84856	1	1	1	1	1	1	1	7
CUX2	1	1	1	1	1	1	1	7
ITPR1	1	1	1	1	1	1	1	7
GFRA1	1	1	1	1	1	1	1	7
KIT	1	1	1	1	1	1	1	7
EFEMP1	1	1	1	1	1	1	1	7
AQP7	1	1	1	0	1	1	1	6
EBF3	1	1	1	0	1	1	1	6
SLC10A4	1	1	1	0	1	1	1	6
C1QTNF9B	1	1	1	0	1	1	1	6
COLEC11	1	1	1	0	1	1	1	6
TM4SF18	1	1	1	0	1	1	1	6
LOC145820	1	1	1	0	1	1	1	6
VIPR1	1	1	1	0	1	1	1	6
SLC14A1	1	1	1	0	1	1	1	6
QRFPR	1	1	1	0	1	1	1	6
NOSTRIN	1	1	1	0	1	1	1	6
CLCNKB	1	1	1	0	1	1	1	6
ALDH1A1	1	1	1	0	1	1	1	6
DNASE1L3	1	1	1	0	1	1	1	6
EMCN	1	1	1	0	1	1	1	6
KY	1	1	1	0	1	1	1	6
PTPRR	1	1	1	0	1	1	1	6
KCNJ13	1	1	1	0	1	1	1	6
CETP	1	1	1	0	1	1	1	6
AGTR1	1	1	1	0	1	1	1	6
C3orf32	1	1	1	0	1	1	1	6

TMEM88	1	1	1	0	1	1	1	6
CLCNKA	1	1	1	0	1	1	1	6
ANO2	1	1	1	0	1	1	1	6
CLEC3B	1	1	1	0	1	1	1	6
CA4	1	1	1	0	1	1	1	6
ROBO4	1	1	1	0	1	1	1	6
EXOC3L2	1	1	1	0	1	1	1	6
SHISA2	1	1	1	0	1	1	1	6
REM1	1	1	1	0	1	1	1	6
KDR	1	1	1	0	1	1	1	6
KCNAB1	1	1	1	0	1	1	1	6
LOC1001302	1	1	1	0	1	1	1	6
LOC90246	1	1	1	0	1	1	1	6
ST3GAL6	1	1	1	0	1	1	1	6
TRPC6	1	1	1	0	1	1	1	6
RTN4RL1	1	1	1	0	1	1	1	6
FLT4	1	1	1	0	1	1	1	6
CCL14	1	1	1	0	1	1	1	6
FHL1	1	1	1	0	1	1	1	6
FAM162B	1	1	1	0	1	1	1	6
FCGBP	1	1	1	0	1	1	1	6
TAL1	1	1	1	0	1	1	1	6
ADRB1	1	1	1	0	1	1	1	6
USHBP1	1	1	1	0	1	1	1	6
CETN4P	1	1	1	0	1	1	1	6
PDE1B	1	1	1	0	1	1	1	6
ANGPTL1	1	1	1	0	1	1	1	6
NMUR1	1	1	1	0	1	1	1	6
MYRIP	1	1	1	0	1	1	1	6
ANGPT1	1	1	1	0	1	1	1	6

DIRAS2	1	1	1	0	1	1	1	6
LRRTM1	1	1	1	0	1	1	1	6
SCN4B	1	1	1	0	1	1	1	6
FRZB	1	1	1	0	1	1	1	6
GPR126	1	1	1	0	1	1	1	6
SLCO1C1	1	1	1	0	1	1	1	6
ESRRG	1	1	1	0	1	1	1	6
MOSC1	1	1	1	0	1	1	1	6
MAB21L2	1	1	1	0	1	1	1	6
PLVAP	1	1	1	0	1	1	1	6
GLDC	1	1	1	0	1	1	1	6
NFASC	1	1	1	0	1	1	1	6
IGDCC4	1	1	1	0	1	1	1	6
SUSD2	1	1	1	0	1	1	1	6
MT1G	1	1	1	0	1	1	1	6
FRMPD1	1	1	1	0	1	1	1	6
LRRC70	1	1	1	0	1	1	1	6
BCO2	1	1	1	0	1	1	1	6
FAM189A1	1	1	1	0	1	1	1	6
CLEC4E	1	1	1	0	1	1	1	6
SCN3A	1	1	1	0	1	1	1	6
SP6	1	1	1	0	1	1	1	6
FMO1	1	1	1	0	1	1	1	6
KCNK17	1	1	1	0	1	1	1	6
LOC286002	1	1	1	0	1	1	1	6
TMEM47	1	1	1	0	1	1	1	6
CASP12	1	1	1	0	1	1	1	6
OCA2	1	1	1	0	1	1	1	6
CABP1	1	1	1	0	1	1	1	6
MT1L	1	1	1	0	1	1	1	6

ANO3	1	1	1	0	1	1	1	6
SGK1	1	1	1	0	1	1	1	6
KITLG	1	1	1	0	1	1	1	6
RSPH4A	1	1	1	0	1	1	1	6
USP44	1	1	1	0	1	1	1	6
LIPG	1	1	1	0	1	1	1	6
AKAP3	1	1	1	0	1	1	1	6
SLC13A4	1	1	1	0	1	1	1	6
CNTN5	1	1	1	0	1	1	1	6
PDE7B	1	1	1	0	1	1	1	6
C9orf24	1	1	1	0	1	1	1	6
ANKK1	1	1	1	0	1	1	1	6
SULT1C4	1	1	1	0	1	1	1	6
PTGDR	1	1	1	0	1	1	1	6
EBF4	1	1	1	0	1	1	1	6
SELV	1	1	1	0	1	1	1	6
RERG	1	1	1	0	1	1	1	6
CDON	1	1	1	0	1	1	1	6
PLCH1	1	1	1	0	1	1	1	6
DIO2	1	1	1	0	1	1	1	6
P2RY8	1	1	1	0	1	1	1	6
MIOX	1	1	1	0	1	1	1	6
CACNB2	1	1	1	0	1	1	1	6
RAPGEF4	1	1	1	0	1	1	1	6
GBP4	1	1	1	0	1	1	1	6
MEOX2	1	1	1	0	1	1	1	6
IPCEF1	1	1	1	0	1	1	1	6
FHL5	1	1	1	0	1	1	1	6
XK	1	1	1	0	1	1	1	6
MYOM1	1	1	1	0	1	1	1	6

C2orf73	1	1	1	0	1	1	1	6
SMAD5OS	1	1	1	0	1	1	1	6
SLC16A2	1	1	1	0	1	1	1	6
GPR98	1	1	1	0	1	1	1	6
LRP2	1	1	1	0	1	1	1	6
SYN3	1	1	1	0	1	1	1	6
SLC26A4	1	1	1	0	1	1	1	6
SHANK2	1	1	1	0	1	1	1	6
MATN2	1	1	1	0	1	1	1	6
AVPR1A	1	1	1	0	1	1	1	6
RBM20	1	1	1	0	1	1	1	6
IL22RA1	1	1	1	0	1	1	1	6
CLEC9A	1	1	1	0	1	1	1	6
MT1F	1	1	1	0	1	1	1	6
TSPAN33	1	1	1	0	1	1	1	6
STXBP5L	1	1	1	0	1	1	1	6
RXFP1	1	1	1	0	1	1	1	6
SLC26A7	1	1	1	0	1	1	1	6
IP6K3	1	1	1	0	1	1	1	6
C15orf51	1	1	1	0	1	1	1	6
ARHGAP28	1	1	1	0	1	1	1	6
LOC201651	1	1	1	0	1	1	1	6
RGS9	1	1	1	0	1	1	1	6
LRP1B	1	1	1	0	1	1	1	6
FAM13C	1	1	1	0	1	1	1	6
RANBP3L	1	1	1	0	1	1	1	6
ELMO1	1	1	1	0	1	1	1	6
LGI3	1	1	1	0	1	1	1	6
SLC5A4	1	1	1	0	1	1	1	6
B3GALT2	1	1	1	0	1	1	1	6

PTPRQ	1	1	1	0	1	1	1	6
CDHR1	1	1	1	0	1	1	1	6
KCNC1	1	1	0	1	1	1	1	6
RIMKLA	1	1	1	0	1	1	1	6
TMEM171	1	1	1	0	1	1	1	6
GRB14	1	1	1	0	1	1	1	6
HOXA2	1	1	1	0	1	1	1	6
SLC1A2	1	1	1	0	1	1	1	6
SORCS1	1	1	1	0	1	1	1	6
ARHGAP24	1	1	1	0	1	1	1	6
DLX1	1	1	1	0	1	1	1	6
PLA1A	1	1	1	0	1	1	1	6
LOC1001281	1	1	1	0	1	1	1	6
MT1H	1	1	1	0	1	1	1	6
MAOB	1	1	1	0	1	1	1	6
KCNJ10	1	1	1	0	1	1	1	6
PTCH2	1	1	1	0	1	1	1	6
KHDRBS2	1	1	1	0	1	1	1	6
RADIL	1	1	1	0	1	1	1	6
INPP5J	1	1	1	0	1	1	1	6
MAPK4	1	1	0	1	1	1	1	6
MLC1	1	1	1	0	1	1	1	6
DHDPSL	1	1	1	0	1	1	1	6
DPY19L2	1	1	1	0	1	1	1	6
MT1M	1	1	1	0	1	1	1	6
LOC400043	1	1	1	0	1	1	1	6
TMEM38A	0	1	1	1	1	1	1	6
L3MBTL4	0	1	1	1	1	1	1	6
AKR1C2	0	1	1	1	1	1	1	6
DPT	0	1	1	1	1	1	1	6

TRPV4	0	1	1	1	1	1	1	6
ANK1	0	1	1	1	1	1	1	6
GPC3	0	1	1	1	1	1	1	6
PRKCB	0	1	1	1	1	1	1	6
PRKCA	0	1	1	1	1	1	1	6
GPR17	1	1	0	0	1	1	1	5
EXOC3L	1	1	0	0	1	1	1	5
EMID1	1	1	0	0	1	1	1	5
TMSB15A	1	1	1	0	1	1	0	5
PCDHB15	1	1	1	0	1	1	0	5
AVPR2	1	1	1	0	1	0	1	5
CD36	1	1	1	0	1	0	1	5
COL9A1	1	1	0	0	1	1	1	5
HS6ST3	1	1	0	0	1	1	1	5
FOXJ1	1	1	0	0	1	1	1	5
GRRP1	1	1	0	0	1	1	1	5
FAM155B	1	1	0	0	1	1	1	5
KATNAL2	1	1	0	0	1	1	1	5
LILRB5	1	1	1	0	1	0	1	5
CCDC48	1	1	1	0	1	1	0	5
COL25A1	1	1	0	0	1	1	1	5
LOC151174	1	1	1	0	0	1	1	5
PYGM	1	1	0	0	1	1	1	5
COLEC10	1	1	0	0	1	1	1	5
C19orf35	1	1	1	0	1	0	1	5
QRICH2	1	1	0	0	1	1	1	5
EFHC2	1	1	0	0	1	1	1	5
C16orf74	1	1	0	0	1	1	1	5
ME1	1	1	0	0	1	1	1	5
WFDC1	1	1	1	0	1	0	1	5

LOC400759	1	1	1	0	0	1	1	5
SCN3B	1	1	0	0	1	1	1	5
AGMAT	1	1	0	0	1	1	1	5
TDO2	1	1	0	0	1	1	1	5
TCF7L1	1	1	0	0	1	1	1	5
SERHL2	1	1	0	0	1	1	1	5
CORO2B	1	1	0	1	1	1	0	5
FOXF2	1	1	0	0	1	1	1	5
PFKFB1	1	1	0	0	1	1	1	5
MYH3	1	1	0	0	1	1	1	5
FGFBP2	1	1	1	0	1	0	1	5
SLC25A34	1	1	0	0	1	1	1	5
BOC	1	1	0	0	1	1	1	5
CACNA2D2	1	1	0	0	1	1	1	5
GPX3	1	1	0	0	1	1	1	5
ZNF323	1	1	1	0	1	1	0	5
FAM167A	1	1	0	1	1	1	0	5
ZNF521	1	1	0	0	1	1	1	5
ZNF541	1	1	0	0	1	1	1	5
SELP	1	1	1	0	1	0	1	5
MYH11	1	1	0	0	1	1	1	5
PAPSS2	0	1	1	1	1	0	1	5
RGNEF	0	1	1	0	1	1	1	5
CR2	0	1	1	0	1	1	1	5
F10	0	1	1	0	1	1	1	5
TNFRSF11B	0	1	0	1	1	1	1	5
HAND2	0	1	1	1	1	1	0	5
MS4A1	0	1	1	0	1	1	1	5
ADAM22	0	1	1	1	1	0	1	5
C9orf122	0	1	1	0	1	1	1	5

LRMP	0	1	1	0	1	1	1	5
BMX	0	1	1	0	1	1	1	5
C6orf165	0	1	1	0	1	1	1	5
AQP4	0	1	1	0	1	1	1	5
ADH1B	0	1	1	1	1	0	1	5
C3orf55	0	1	1	1	1	0	1	5
PREX2	0	1	1	1	1	0	1	5
CD27	0	1	1	0	1	1	1	5
LRRC2	0	1	1	0	1	1	1	5
DPY19L2P4	0	1	1	0	1	1	1	5
FREM2	0	1	1	0	1	1	1	5
ABCG2	1	1	1	0	1	0	0	4
GPER	1	1	0	0	1	1	0	4
C13orf15	1	1	0	0	1	1	0	4
HRC	1	1	1	0	1	0	0	4
KCNIP3	1	1	0	0	1	1	0	4
TSPAN7	1	1	0	0	1	1	0	4
DUOXA2	1	1	0	0	0	1	1	4
ASCL2	1	1	0	0	0	1	1	4
LOC728264	1	1	0	0	1	1	0	4
THSD7B	1	1	0	0	1	1	0	4
ATP1B2	1	1	0	0	1	1	0	4
C14orf50	1	1	0	0	0	1	1	4
GPR83	1	1	0	0	1	1	0	4
PKNOX2	1	1	0	0	1	1	0	4
EDA	1	1	0	0	1	1	0	4
DEPDC6	1	1	0	0	1	1	0	4
FAM181B	1	1	0	0	1	1	0	4
OXCT2	1	1	0	0	1	1	0	4
FABP4	1	1	0	0	1	0	1	4

FBXO15	1	1	0	0	1	1	0	4
SLC5A5	1	1	1	0	1	0	0	4
SLC29A4	1	1	0	0	1	1	0	4
C16orf89	1	1	0	0	1	1	0	4
FGF12	1	1	0	0	1	1	0	4
TMC3	1	1	0	0	1	1	0	4
SDF2L1	1	1	0	0	1	1	0	4
TSPAN18	1	1	0	0	1	1	0	4
GZMM	1	1	1	0	0	0	1	4
WDR63	1	1	0	0	1	0	1	4
JPH4	1	1	0	0	1	0	1	4
EYA4	1	1	0	0	1	1	0	4
CLMN	1	1	0	0	1	1	0	4
PPP1R3C	1	1	0	1	1	0	0	4
CPNE5	1	1	0	0	1	0	1	4
TDH	1	1	0	0	1	1	0	4
FGD2	1	1	0	0	1	0	1	4
TPO	0	1	0	0	1	1	1	4
BMP8A	0	1	0	0	1	1	1	4
MYCN	0	1	1	0	1	0	1	4
FMO2	0	1	1	0	1	0	1	4
KCNA3	0	1	1	0	1	0	1	4
ATRNL1	0	1	0	0	1	1	1	4
MASP1	0	1	1	0	1	0	1	4
DDIT4L	0	1	0	1	1	1	0	4
ASXL3	0	1	0	0	1	1	1	4
SPARCL1	0	1	1	0	1	0	1	4
PID1	0	1	1	0	1	0	1	4
GPR37L1	0	1	0	0	1	1	1	4
UPF0639	0	1	1	0	1	1	0	4

LRRC4C	0	1	0	0	1	1	1	4
ANKRD29	0	1	1	0	1	0	1	4
C12orf39	0	1	0	0	1	1	1	4
C7orf58	0	1	1	0	1	0	1	4
RTN1	0	1	0	1	1	0	1	4
COCH	0	1	0	1	1	0	1	4
CACNA2D3	0	1	0	0	1	1	1	4
CCDC68	0	1	0	1	1	0	1	4
C9orf152	0	1	0	0	1	1	1	4
NCF1B	0	1	1	0	1	0	1	4
AR	0	1	0	0	1	1	1	4
RBP5	0	1	0	0	1	1	1	4
FAM3D	1	1	0	0	0	1	0	3
ROPN1B	1	1	0	0	1	0	0	3
GALNT14	1	1	0	0	1	0	0	3
DLX6	1	1	0	0	0	1	0	3
GLIPR1L2	1	1	0	0	0	1	0	3
TXNDC6	1	1	0	0	0	1	0	3
TMEM90B	1	1	0	0	1	0	0	3
FLT1	1	1	0	0	1	0	0	3
DNALI1	1	1	0	0	0	1	0	3
BEGAIN	1	1	0	0	1	0	0	3
COL9A3	1	1	0	0	1	0	0	3
C3orf36	1	1	0	0	1	0	0	3
PURG	1	1	0	0	1	0	0	3
COX7A1	1	1	0	0	1	0	0	3
TBX21	1	1	0	0	0	0	1	3
AOC3	1	1	0	0	1	0	0	3
EDN2	1	1	0	0	1	0	0	3
KLF2	1	1	0	0	1	0	0	3

DACH2	1	1	0	0	0	1	0	3
NPR1	1	1	0	0	1	0	0	3
AKR1C1	0	1	0	0	1	1	0	3
OSBPL6	0	1	0	0	1	1	0	3
SLC17A7	0	1	0	0	1	1	0	3
OLFM2	0	1	0	0	1	1	0	3
GCSH	0	1	0	0	1	1	0	3
LRRC55	0	1	0	0	1	0	1	3
SSPO	0	1	0	0	1	1	0	3
EPB41L3	0	1	0	0	1	0	1	3
SH2D6	0	1	1	0	1	0	0	3
CAPSL	0	1	0	0	1	1	0	3
EOMES	0	1	1	0	0	0	1	3
C1QTNF2	0	1	0	0	1	1	0	3
TSNAXIP1	0	1	0	0	1	1	0	3
C8orf84	0	1	0	0	1	0	1	3
LOC283174	0	1	0	0	1	1	0	3
COL14A1	0	1	0	0	1	1	0	3
COL4A6	0	1	0	0	1	1	0	3
KCNK2	0	1	0	0	1	1	0	3
CDCA7	0	1	0	0	1	0	1	3
NRG2	0	1	0	0	1	1	0	3
TRPV6	0	1	0	0	1	1	0	3
ABCC8	0	1	0	0	0	1	1	3
ITGA8	0	1	0	1	1	0	0	3
TPPP	0	1	0	0	1	1	0	3
CECR1	0	1	0	0	0	1	1	3
INHBC	0	1	1	0	1	0	0	3
NOL4	0	1	0	0	1	1	0	3
ANKRD20B	0	1	0	0	1	1	0	3

SSTR1	0	1	0	0	1	1	0	3
UGT8	0	1	0	1	1	0	0	3
GOLGA9P	0	1	0	0	1	1	0	3
LOC1001297	0	1	0	0	1	1	0	3
PKDCC	0	1	0	0	1	1	0	3
ADCY1	0	1	0	1	1	0	0	3
SLC6A17	0	1	0	1	1	0	0	3
NPL	0	1	0	0	1	0	1	3
C1orf127	0	1	0	0	1	1	0	3
MMRN1	0	1	0	0	1	0	1	3
DNAH9	0	1	0	0	1	1	0	3
PROX1	0	1	0	1	1	0	0	3
LOC342346	0	1	0	0	1	1	0	3
CDYL2	0	1	0	1	1	0	0	3
C10orf79	0	1	0	0	1	1	0	3
FGL2	0	1	0	0	1	0	1	3
CYP46A1	0	1	0	0	1	1	0	3
FAM26F	0	1	1	0	0	0	1	3
FAM95B1	0	1	0	0	1	1	0	3
DOC2A	0	0	0	0	1	1	1	3
DLEU7	0	0	0	0	1	1	1	3
HIGD1B	1	1	0	0	0	0	0	2
TBXA2R	1	1	0	0	0	0	0	2
NMUR2	1	1	0	0	0	0	0	2
SDPR	1	1	0	0	0	0	0	2
RGS5	1	1	0	0	0	0	0	2
SCG3	1	1	0	0	0	0	0	2
ST6GALNAC1	1	1	0	0	0	0	0	2
VAV3	1	0	0	0	0	0	1	2
SUCNR1	0	1	0	0	1	0	0	2

SGCD	0	1	0	0	1	0	0	2
FBXO17	0	1	0	0	1	0	0	2
ACSL6	0	1	0	0	1	0	0	2
CYP7B1	0	1	0	0	1	0	0	2
ART5	0	1	0	0	0	0	1	2
BLK	0	1	0	0	1	0	0	2
HOXA3	0	1	0	0	1	0	0	2
LRRN2	0	1	0	0	0	1	0	2
HKDC1	0	1	0	0	1	0	0	2
LMOD1	0	1	0	0	1	0	0	2
MAB21L1	0	1	0	0	1	0	0	2
CSMD2	0	1	0	0	1	0	0	2
CYP26B1	0	1	0	0	1	0	0	2
CDHR3	0	1	0	0	1	0	0	2
PRIMA1	0	1	0	0	1	0	0	2
GGTLC1	0	1	0	0	0	1	0	2
SCUBE2	0	1	0	0	1	0	0	2
BVES	0	1	0	0	1	0	0	2
HS3ST3B1	0	1	0	0	1	0	0	2
DHH	0	1	0	0	1	0	0	2
SLITRK5	0	1	0	0	1	0	0	2
SUSD5	0	1	0	0	1	0	0	2
SLC24A3	0	1	0	0	1	0	0	2
ROPN1L	0	1	0	0	1	0	0	2
LGI4	0	1	0	0	1	0	0	2
PCDP1	0	1	0	0	1	0	0	2
PLAC9	0	1	0	0	1	0	0	2
HECW2	0	1	0	0	1	0	0	2
SMOC2	0	1	0	0	1	0	0	2
PCBP3	0	1	0	0	1	0	0	2

ANK2	0	1	0	0	1	0	0	2
EDN3	0	1	0	0	0	1	0	2
ART4	0	1	0	0	1	0	0	2
AOC2	0	1	0	0	1	0	0	2
C1QTNF7	0	1	0	0	1	0	0	2
GRAPL	0	1	0	0	1	0	0	2
SCUBE1	0	1	0	0	1	0	0	2
HSPB6	0	1	0	0	1	0	0	2
RNF150	0	1	0	0	1	0	0	2
NEB	0	1	0	0	1	0	0	2
TCEAL2	0	1	0	0	0	1	0	2
RFPL1S	0	1	0	1	0	0	0	2
AKR1E2	0	1	0	0	1	0	0	2
OTOS	0	1	0	0	1	0	0	2
FLJ35024	0	1	0	0	1	0	0	2
IL33	0	1	0	0	1	0	0	2
EPHA3	0	1	0	0	1	0	0	2
DENND2A	0	1	0	0	1	0	0	2
C1QTNF9	0	1	0	0	1	0	0	2
SYBU	0	1	0	0	1	0	0	2
BTBD11	0	1	0	0	1	0	0	2
CHST8	0	1	0	0	1	0	0	2
PPM1L	0	1	0	0	1	0	0	2
TRPM6	0	1	0	0	1	0	0	2
FMOD	0	1	0	0	1	0	0	2
FAM166B	0	1	0	0	0	1	0	2
TPTE2P1	0	1	0	0	0	1	0	2
BCL11B	0	1	0	0	1	0	0	2
C9orf66	0	1	0	0	0	1	0	2
CHRD	0	1	0	0	1	0	0	2

ENPP3	0	1	0	0	0	1	0	2
GLT8D2	0	1	0	0	1	0	0	2
KIF1A	0	1	0	0	1	0	0	2
DOCK10	0	1	0	0	0	0	1	2
LRFN1	0	1	0	0	1	0	0	2
ABCC4	0	1	0	0	1	0	0	2
GNAL	0	1	0	0	1	0	0	2
EDARADD	0	1	0	0	1	0	0	2
C20orf26	0	1	0	0	1	0	0	2
PDE11A	0	1	0	0	1	0	0	2
PTGDS	0	1	0	0	1	0	0	2
FAM129C	0	1	0	0	1	0	0	2
C10orf128	0	1	0	0	1	0	0	2
DNAJC12	0	1	0	0	1	0	0	2
ST8SIA1	0	1	0	0	1	0	0	2
RASGRP2	0	1	0	0	1	0	0	2
ATP6V1G2	0	1	0	0	0	1	0	2
CPXM2	0	1	0	0	1	0	0	2
ISLR2	0	1	0	0	1	0	0	2
HRH2	0	0	0	1	1	0	0	2
FLRT2	0	0	0	0	1	1	0	2
GYG2	0	0	0	0	1	1	0	2
FAM19A1	0	0	0	0	1	1	0	2
CD163L1	0	0	0	0	1	0	1	2
PLB1	0	0	0	0	1	0	1	2
ANKRD20A3	0	0	0	0	1	1	0	2
GPR62	0	0	0	0	0	1	1	2
CX3CR1	1	0	0	0	0	0	0	1
HCRTR1	1	0	0	0	0	0	0	1
LOC134466	1	0	0	0	0	0	0	1

CKMT2	1	0	0	0	0	0	0	1
ASPA	1	0	0	0	0	0	0	1
LIN7A	1	0	0	0	0	0	0	1
NDUFA4L2	1	0	0	0	0	0	0	1
SYT6	1	0	0	0	0	0	0	1
COX4I2	1	0	0	0	0	0	0	1
RNF157	1	0	0	0	0	0	0	1
DUSP27	1	0	0	0	0	0	0	1
MYCBPAP	1	0	0	0	0	0	0	1
ABCG4	1	0	0	0	0	0	0	1
SLC4A5	0	1	0	0	0	0	0	1
CD8A	0	1	0	0	0	0	0	1
VPREB3	0	1	0	0	0	0	0	1
DCLK3	0	1	0	0	0	0	0	1
GALNT9	0	1	0	0	0	0	0	1
BST2	0	1	0	0	0	0	0	1
WDR65	0	1	0	0	0	0	0	1
PRR18	0	1	0	0	0	0	0	1
LHFPL1	0	1	0	0	0	0	0	1
TOX	0	1	0	0	0	0	0	1
MT1E	0	1	0	0	0	0	0	1
ITPRIPL1	0	1	0	0	0	0	0	1
SGCA	0	1	0	0	0	0	0	1
MUSTN1	0	1	0	0	0	0	0	1
CCDC141	0	1	0	0	0	0	0	1
LOC389493	0	1	0	0	0	0	0	1
AGAP2	0	1	0	0	0	0	0	1
CMKLR1	0	1	0	0	0	0	0	1
PBX4	0	1	0	0	0	0	0	1
PRF1	0	1	0	0	0	0	0	1

PAH	0	1	0	0	0	0	0	1
SAMD3	0	1	0	0	0	0	0	1
C12orf69	0	1	0	0	0	0	0	1
NKG7	0	1	0	0	0	0	0	1
FCN3	0	1	0	0	0	0	0	1
RUNDC2A	0	0	0	1	0	0	0	1
KLHL11	0	0	0	1	0	0	0	1
FADS2	0	0	0	1	0	0	0	1
LOC402377	0	0	0	1	0	0	0	1
PLXNB3	0	0	0	0	1	0	0	1
NDRG4	0	0	0	0	1	0	0	1
ROR2	0	0	0	0	1	0	0	1
DGKI	0	0	0	0	1	0	0	1
MRGPRF	0	0	0	0	1	0	0	1
IGF2BP3	0	0	0	0	1	0	0	1
FOXF1	0	0	0	0	1	0	0	1
WNT11	0	0	0	0	1	0	0	1
CECR2	0	0	0	0	1	0	0	1
PCDHB18	0	0	0	0	1	0	0	1
OSR1	0	0	0	0	1	0	0	1
ADAM33	0	0	0	0	1	0	0	1
NLGN3	0	0	0	0	1	0	0	1
RPS6KL1	0	0	0	0	1	0	0	1
GPR20	0	0	0	0	1	0	0	1
PSAT1	0	0	0	0	1	0	0	1
CILP	0	0	0	0	1	0	0	1
COPZ2	0	0	0	0	1	0	0	1
TLL1	0	0	0	0	1	0	0	1
C9orf43	0	0	0	0	1	0	0	1
TMEM63C	0	0	0	0	1	0	0	1

CHRNA3	0	0	0	0	1	0	0	1
CD300C	0	0	0	0	1	0	0	1
STEAP2	0	0	0	0	1	0	0	1
CCDC85A	0	0	0	0	1	0	0	1
CACNA1H	0	0	0	0	1	0	0	1
BCL2L15	0	0	0	0	1	0	0	1
ZFPM2	0	0	0	0	1	0	0	1
ADM2	0	0	0	0	1	0	0	1
ADAMTS5	0	0	0	0	1	0	0	1
FXYD1	0	0	0	0	1	0	0	1
PDE10A	0	0	0	0	1	0	0	1
MGAT5B	0	0	0	0	1	0	0	1
SMTNL2	0	0	0	0	1	0	0	1
OLFML1	0	0	0	0	1	0	0	1
RASSF6	0	0	0	0	1	0	0	1
PGF	0	0	0	0	1	0	0	1
PPARGC1A	0	0	0	0	1	0	0	1
KLHL4	0	0	0	0	1	0	0	1
PGM5P2	0	0	0	0	1	0	0	1
FHL2	0	0	0	0	1	0	0	1
WFIKKN1	0	0	0	0	1	0	0	1
FABP5	0	0	0	0	1	0	0	1
C4orf31	0	0	0	0	1	0	0	1
EPHB1	0	0	0	0	1	0	0	1
RBM24	0	0	0	0	1	0	0	1
GRAMD2	0	0	0	0	1	0	0	1
AKAP12	0	0	0	0	1	0	0	1
SIGLEC11	0	0	0	0	1	0	0	1
PDPN	0	0	0	0	1	0	0	1
ITIH3	0	0	0	0	1	0	0	1

PLA2G4F	0	0	0	0	1	0	0	1
LRRN4CL	0	0	0	0	1	0	0	1
ISM1	0	0	0	0	1	0	0	1
C12orf68	0	0	0	0	1	0	0	1
STK32B	0	0	0	0	1	0	0	1
HGF	0	0	0	0	1	0	0	1
MYOM3	0	0	0	0	1	0	0	1
PCSK1	0	0	0	0	1	0	0	1
MGC42105	0	0	0	0	1	0	0	1
VIP	0	0	0	0	0	1	0	1
C18orf16	0	0	0	0	0	1	0	1
EFHB	0	0	0	0	0	1	0	1
C20orf203	0	0	0	0	0	1	0	1
PACSIN1	0	0	0	0	0	0	1	1
GPR34	0	0	0	0	0	0	1	1
TNFSF8	0	0	0	0	0	0	1	1

*IMRT: Treated with Intensity Modulated Radiation Therapy

**Patient displayed persistent loco-regional or distant disease

***Patient had tumor at follow-up

****New tumor event after initial pathological diagnosis

*****Number of left-out groups for which gene remained significantly differentially regulated

Supplementary table 5. Genes downregulated in poor-prognosis PTC & overlapping with published (referenced) gene-sets

Gene	Reyes_markers (ref #23)	Dely_markers (ref 20)	Griffith_markers (ref #18)	TSgene_Tumor_Suppressor_Genes (ref #16)
ACE	ACE		NA	NA
AOX1	AOX1	AOX1	NA	NA
ATP2C2	ATP2C2		NA	NA
AXIN2	NA	NA	NA	AXIN2
CCL21		CCL21	NA	NA
CDH16	CDH16	CDH16	NA	NA
CXCL12		CXCL12	NA	NA
DIO1		DIO1	DIO1	NA
DLG2		DLG2	NA	NA
DPP6	DPP6	DPP6	NA	NA
FLRT1	FLRT1		NA	NA
GABRB3	GABRB3		NA	NA
GDF10		GDF10	NA	NA
GFRA1	GFRA1		NA	NA
GNA14	GNA14	GNA14	NA	NA
GPMBA	GPMBA		NA	NA
HGD	HGD	HGD	NA	NA
IGFBPL1	IGFBPL1		NA	IGFBPL1
IQGAP2		IQGAP2	NA	IQGAP2
IRS1		IRS1	NA	NA
ITPR1	ITPR1	ITPR1	ITPR1	NA
KIAA1324	KIAA1324		NA	NA
KIT		KIT	NA	NA
LIFR	LIFR	LIFR	NA	LIFR
LTF		LTF	NA	LTF
MPPED2		MPPED2	NA	NA
PLA2R1	PLA2R1	PLA2R1	NA	NA
RASL11A	NA	NA	NA	RASL11A
RASSF9	RASSF9	RASSF9	NA	NA
RELN		RELN	NA	NA
RERGL		RERGL	NA	NA
RYR2	RYR2	RYR2	NA	NA
SEMA3D		SEMA3D	NA	NA
SLC4A4	SLC4A4	SLC4A4	NA	NA
SLC5A8	NA	NA	NA	SLC5A8
SORBS2		SORBS2	NA	NA
SYNE1		SYNE1	NA	NA
SYNM		SYNM	NA	SYNM
TFCP2C1	TFCP2C1		NA	NA
TFF3	NA	NA	TFF3	NA
WSCD2	WSCD2		NA	NA
ZNF366	NA	NA	NA	ZNF366

Supplementary Table 6: Distribution of clinicopathological and demographic factors in intermediate risk patients classified as good or poor prognosis by PAM model (All samples)

Factor	Level	Poor Prognosis (n)	Good Prognosis (n)	P-value* (difference between prognostic groups)
Extra-thyroid extension	None	97	111	
	Minimal	46	19	
	Moderate/Advanced	1	0	0.001
Nodal involvement	Absent	63	74	
	Present	94	40	<0.0001
Clinical stage	Stage 1	40	30	
	Stage 2	72	70	
	Stage 3	64	35	0.1
Mutations	BRAF	165	26	<0.0001
	HRAS	0	11	<0.0001
	KRAS	1	2	0.67
	NRAS	6	19	<0.0001
Histological subtype	Classical	177	77	
	Follicular	16	62	
	Tall Cell	28	3	<0.0001
Age at diagnosis	(Mean (Inter-quartile range))	45.4 (33-56)	47.66 (34.5-60)	0.17

Gender	Female	163	108	
	Male	64	35	0.51
Race	Asian	22	13	
	Black/African	11	3	
	White	158	71	0.55

*(Pearson chi-squared (categorical variables), or Student's T-test (continous variables)

Supplementary table 7: Distribution of clinical characteristics of intermediate good and intermediate poor prognosis groups in leave-one-out cross validation

Left-out-group	Intermediate good prognosis		Intermediate poor prognosis	
	**Performance (number of poor prognosis patients assigned to group)**			
	Number	Percentage	Number	Percentage
Adjuvant drug therapy	6	0.38	10	0.63
IMRT*	1	0.5	1	0.5
Persistent disease**	3	0.27	8	0.73
With tumor***	18	0.43	24	0.57
Died of PTC	5	0.36	9	0.64
Metastases present	1	0.11	8	0.89
New tumor event****	2	0.2	8	0.8
	**Mutation (percentage of samples with)**			
	BRAF	RASx*	BRAF	RASx*
Adjuvant drug therapy	0.1	0.09	0.36	0.01
IMRT	0.06	0.08	0.37	0.01
Persistent disease	0.08	0.08	0.36	0.01
With tumor	0.19	0.09	0.33	0.01
Died of PTC	0.08	0.08	0.36	0.01
Metastases present	0.06	0.09	0.36	0.01
New tumor event	0.07	0.08	0.36	0.01
	**Nodal involvement (n)**			
	Without	With	Without	With
Adjuvant drug therapy	93	46	44	83
IMRT	73	37	44	83
Persistent disease	77	43	44	83
With tumor	99	72	44	83

Died of PTC	77	43			44	83		
Metastases present	78	41			44	83		
New tumor event	74	40			44	83		
	**Stage (n)**							
	Stage 1	Stage 2	Stage 3		Stage 1	Stage 2	Stage 3	
Adjuvant drug therapy	38	80	44		32	58	54	
IMRT	29	67	35		32	58	54	
Persistent disease	36	69	37		32	58	54	
With tumor	51	88	58		32	58	54	
Died of PTC	35	71	36		32	58	54	
Metastases present	32	71	37		32	58	54	
New tumor event	30	70	35		32	58	54	
	**Histological subtype (n)**							
	Unknown	Classical	Follicular	Tall Cell	Unknown	Classical	Follicular	Tall Cell
Adjuvant drug therapy	2	96	68	1	5	144	9	26
IMRT	1	72	61	3	5	144	9	26
Persistent disease	3	80	64	3	5	144	9	26
With tumor	4	142	66	8	5	144	9	26
Died of PTC	2	85	64	4	5	144	9	26
Metastases present	2	78	65	2	5	144	9	26
New tumor event	1	77	62	3	5	144	9	26

*IMRT: Treated with Intensity Modulated Radiation Therapy

**Patient displayed persistent loco-regional or distant disease

***Patient had tumor at follow-up

****New tumor event after initial pathological diagnosis

*****Number of left-out groups for which gene remained significantly differentially regulated

****** KRAS or HRAS mutations

Supplementary table 8: Genes used within the PAM classifier, and presence (1) or absence (0) of each gene within classifier, when patients representing each of 6 poor prognostic factors are left out

Genes	Left out group (patients associated with specified poor prognositic clinical factor)							
	Adjuvant drug therapy	IMRT**	Persistent disease**	With tumor***	Died of PTC	Metastases present	New tumor event****	Sum*****
WSCD2	1	1	1	1	1	1	1	7
MRO	1	1	1	1	1	1	1	7
TDGF1	1	1	1	1	1	1	1	7
LTF	1	1	1	1	1	1	1	7
LIMS3	1	1	1	1	1	1	1	7
FAM65C	1	1	1	1	1	1	1	7
SOD3	1	1	1	1	1	1	1	7
GRIP2	1	1	1	1	1	1	1	7
RUNX1T1	1	1	1	1	1	1	1	7
CELF4	1	1	1	1	1	1	1	7
PCSK6	1	1	1	1	1	1	1	7
ABCB1	1	1	1	1	1	1	1	7
SYT15	1	1	1	1	1	1	1	7
NR1D1	1	1	1	1	1	1	1	7
PEAR1	1	1	1	1	1	1	1	7
MAP2K6	1	1	1	1	1	1	1	7
RCAN2	1	1	1	1	1	1	1	7
FAM124B	1	1	1	1	1	1	1	7
GRIN2C	1	1	1	0	1	1	1	6
TFF3	1	1	1	0	1	1	1	6
SPINK5	1	1	1	0	1	1	1	6
KCNJ13	1	1	1	0	1	1	1	6
MPPED2	1	1	1	0	1	1	1	6
FLRT1	1	1	1	0	1	1	1	6
.OC10013023	1	1	1	0	1	1	1	6
KIF19	1	1	1	0	1	1	1	6
GABRB3	1	1	1	0	1	1	1	6
RERGL	1	1	1	1	0	1	1	6
CD300LG	1	1	1	1	1	0	1	6
IGDCC3	1	1	1	0	1	1	1	6
GCK	1	1	1	0	1	1	1	6
IQGAP2	1	1	1	0	1	1	1	6
AQP7	1	1	1	0	1	1	1	6
GNA14	1	1	1	0	1	1	1	6
SLC10A4	1	1	1	0	1	1	1	6
CLCNKB	1	1	1	0	1	1	1	6
FZD9	1	1	1	0	1	1	1	6
REEP1	1	1	1	0	1	1	1	6
CGNL1	1	1	1	0	1	1	1	6
LRRC4B	1	1	1	0	1	1	1	6
FSTL3	1	1	1	0	1	1	1	6
PGM5	1	1	1	0	1	1	1	6
LOC145820	1	1	1	0	1	1	1	6
PTPRR	1	1	1	0	1	1	1	6
SNX22	1	1	1	0	1	1	1	6
EBF3	1	1	1	0	1	1	1	6
PAMR1	1	1	1	0	1	1	1	6
SYNM	1	1	1	0	1	1	1	6
SYNGR1	1	1	1	0	1	1	1	6
TM4SF18	1	1	1	0	1	1	1	6
LRRC4	1	1	1	0	1	1	1	6
EMCN	1	1	1	0	1	1	1	6
NOSTRIN	1	1	1	0	1	1	1	6
CCL21	0	1	1	1	1	1	1	6
CDH16	0	1	1	1	1	1	1	6
PLA2R1	0	1	1	1	1	1	1	6
NOX5	1	1	1	0	0	1	1	5
GRIK4	1	1	0	0	1	1	1	5
AREG	1	1	1	0	0	1	1	5
VIPR1	1	1	1	0	0	1	1	5
KY	1	1	1	0	0	1	1	5
SLC14A1	1	1	1	0	0	1	1	5
C1QTNF9B	1	1	1	0	0	1	1	5
SDK2	1	1	0	1	1	1	0	5
IQGAP3	1	1	1	0	0	1	1	5
SORBS2	1	1	0	0	1	1	1	5
SHISA2	1	1	1	0	0	1	1	5
RASL11A	1	1	1	0	0	1	1	5

SAMD5	0	1	1	1	1	1	0	5
SYNE1	0	1	1	0	1	1	1	5
CLCNKA	1	0	0	0	1	1	1	4
EPHA10	1	0	1	0	0	1	1	4
COLEC11	1	1	1	0	0	1	0	4
C3orf32	1	1	1	0	0	0	1	4
C8orf80	0	1	1	1	0	0	1	4
NWD1	0	1	1	0	1	1	0	4
KIAA1324	0	1	1	0	0	1	1	4
DLG2	0	1	1	1	1	0	0	4
FCGBP	0	1	1	0	1	1	0	4
MYRIP	0	1	1	0	1	0	1	4
AXIN2	0	1	0	1	0	1	1	4
AGTR1	1	1	1	0	0	0	0	3
CCL14	1	0	1	0	1	0	0	3
ANO2	1	0	1	0	0	0	1	3
ALDH1A1	1	0	0	0	0	1	1	3
GDF10	0	0	1	1	1	0	0	3
FAM163A	0	0	1	1	1	0	0	3
GVIN1	0	0	1	1	0	0	1	3
DUSP26	0	0	0	1	0	1	1	3
ADCY5	0	0	0	1	1	1	0	3
IRS1	0	0	0	0	1	1	1	3
KCNAB1	1	0	0	0	0	1	0	2
HIST1H1D	1	0	1	0	0	0	0	2
ST3GAL6	1	0	0	0	0	1	0	2
TRPC6	1	0	0	0	0	1	0	2
ACE	1	0	0	0	1	0	0	2
B3GNT4	1	0	1	0	0	0	0	2
LOC90246	1	0	0	0	1	0	0	2
FAM124A	0	1	0	0	1	0	0	2
RAG1	0	1	0	0	1	0	0	2
SLC5A8	0	0	1	1	0	0	0	2
LOC286002	0	0	1	0	0	1	0	2
CLEC4E	0	0	1	0	0	0	1	2
TFCP2L1	0	0	1	0	1	0	0	2
CNTN2	0	0	1	0	0	1	0	2
UCN	0	0	1	1	0	0	0	2
AOX1	0	0	0	1	1	0	0	2
LRRTM1	0	0	0	0	1	1	0	2
FHL1	0	0	0	0	1	1	0	2
RADIL	0	0	0	0	1	1	0	2
ESRRG	0	0	0	0	1	1	0	2
FLJ23867	0	0	0	0	0	1	1	2
CA4	1	0	0	0	0	0	0	1
LOX	1	0	0	0	0	0	0	1
QRFPR	1	0	0	0	0	0	0	1
CETP	1	0	0	0	0	0	0	1
DNASE1L3	1	0	0	0	0	0	0	1
EXOC3L2	1	0	0	0	0	0	0	1
TMEM88	1	0	0	0	0	0	0	1
REM1	1	0	0	0	0	0	0	1
RTN4RL1	1	0	0	0	0	0	0	1
KDR	1	0	0	0	0	0	0	1
HSN2	1	0	0	0	0	0	0	1
CLEC3B	1	0	0	0	0	0	0	1
FLT4	1	0	0	0	0	0	0	1
ROBO4	1	0	0	0	0	0	0	1
HIST1H1E	0	0	1	0	0	0	0	1
ANGPT1	0	0	1	0	0	0	0	1
KCNH1	0	0	1	0	0	0	0	1
VWF	0	0	1	0	0	0	0	1
GFRA1	0	0	0	1	0	0	0	1
ECEL1	0	0	0	1	0	0	0	1
ITGA8	0	0	0	1	0	0	0	1
HAP1	0	0	0	1	0	0	0	1
.OC10030265	0	0	0	1	0	0	0	1
CXCL12	0	0	0	1	0	0	0	1
ZNF366	0	0	0	1	0	0	0	1
COCH	0	0	0	1	0	0	0	1
.OC10013486	0	0	0	1	0	0	0	1
LOC442421	0	0	0	1	0	0	0	1
CDYL2	0	0	0	1	0	0	0	1

PKHD1L1	0	0	0	0	1	0	0	1
ZMAT4	0	0	0	0	1	0	0	1
SEMA3D	0	0	0	0	1	0	0	1
PI16	0	0	0	0	1	0	0	1
RELN	0	0	0	0	1	0	0	1
IGFBPL1	0	0	0	0	1	0	0	1
RYR2	0	0	0	0	1	0	0	1
KLK10	0	0	0	0	1	0	0	1
ATP2C2	0	0	0	0	1	0	0	1
DIRAS2	0	0	0	0	1	0	0	1
RASSF9	0	0	0	0	1	0	0	1
PAPSS2	0	0	0	0	1	0	0	1
MT1G	0	0	0	0	0	1	0	1
FAM189A1	0	0	0	0	0	1	0	1
APBA2	0	0	0	0	0	1	0	1
LEMD1	0	0	0	0	0	1	0	1
CACNB4	0	0	0	0	0	1	0	1
RSPH4A	0	0	0	0	0	1	0	1
PTCH2	0	0	0	0	0	1	0	1
NFASC	0	0	0	0	0	1	0	1
GLDC	0	0	0	0	0	0	1	1
SIDT1	0	0	0	0	0	0	1	1
C1orf51	0	0	0	0	0	0	1	1

tensity Modulated Radiation Therapy
istent loco-regional or distant disease
: had tumor at follow-up
t after initial pathological diagnosis
:h gene remained significantly differentially regulated
