# Supplementary material for: Development of prognostic signatures for intermediate-risk papillary thyroid cancer
Source: BMC Cancer. 2016 Sep 15;16:736. doi: 10.1186/s12885-016-2771-6 (PMC5025616; doi:10.1186/s12885-016-2771-6)
Supplement: Additional file 2: Figure S1. — MACIS score in extreme prognsos and intermediate prognosis patient groups: MACIS score (based on the presence of distant Metastases, patient Age, Completeness of tumor resection, presence of local Invasion, and tumor Size) was higher in poor prognosis patients (n = 77) than good prognosis patients (n = 50) within the Extreme prognosis patient (training set) patient group (p = 4.28e-07). However, there was no difference in MACIS score between patients predicted as poor (n = 189) and good prognosis (n = 146) witin the medium prognosis (test set) patient group (p = 0.2). This indicates that the MACIS score, indicating that the MACIS score does not have ability to predict patient prognosis as determined by our gene expression classifier. Figure S2. Checking the robustness of the PAM model gene signature: Leaving out one of 7 extreme prognosis patient groups in each round, and using the remaining 6 patient groups to train a Prediction Analysis of Microarrays (PAM) model, the performance of the PAM model in correctly classifying patients within the left-out group to either the good or poor prognosis groups, was tested. ROC curves illustrate the performances of the models for each left out group. Left out groups represent extreme poor prognosis patients, each group associated with a specific poor prognostic clinical factor. Figure S3. Negative correlation between NR1D1 and the overlapping thyroid hormone receptor gene THRA. There was a significant Pearson correlation of expression of NR1D1 and THRA (measured using RNA-Seq) in tumor (cor = −0.09, p = 0.04, n = 501), but not within a smaller set of normal adjacent tissue samples (cor = −0.16, p = 0.24, n = 58). NR1D1 was the gene most strongly upregulated in poor prognosis PTC patients relative to good prognosis patients, and may influence thyroid cancer through downregulation of THRA. (PPTX 1109 kb) [file 12885_2016_2771_MOESM2_ESM.pptx]

## Slide 1
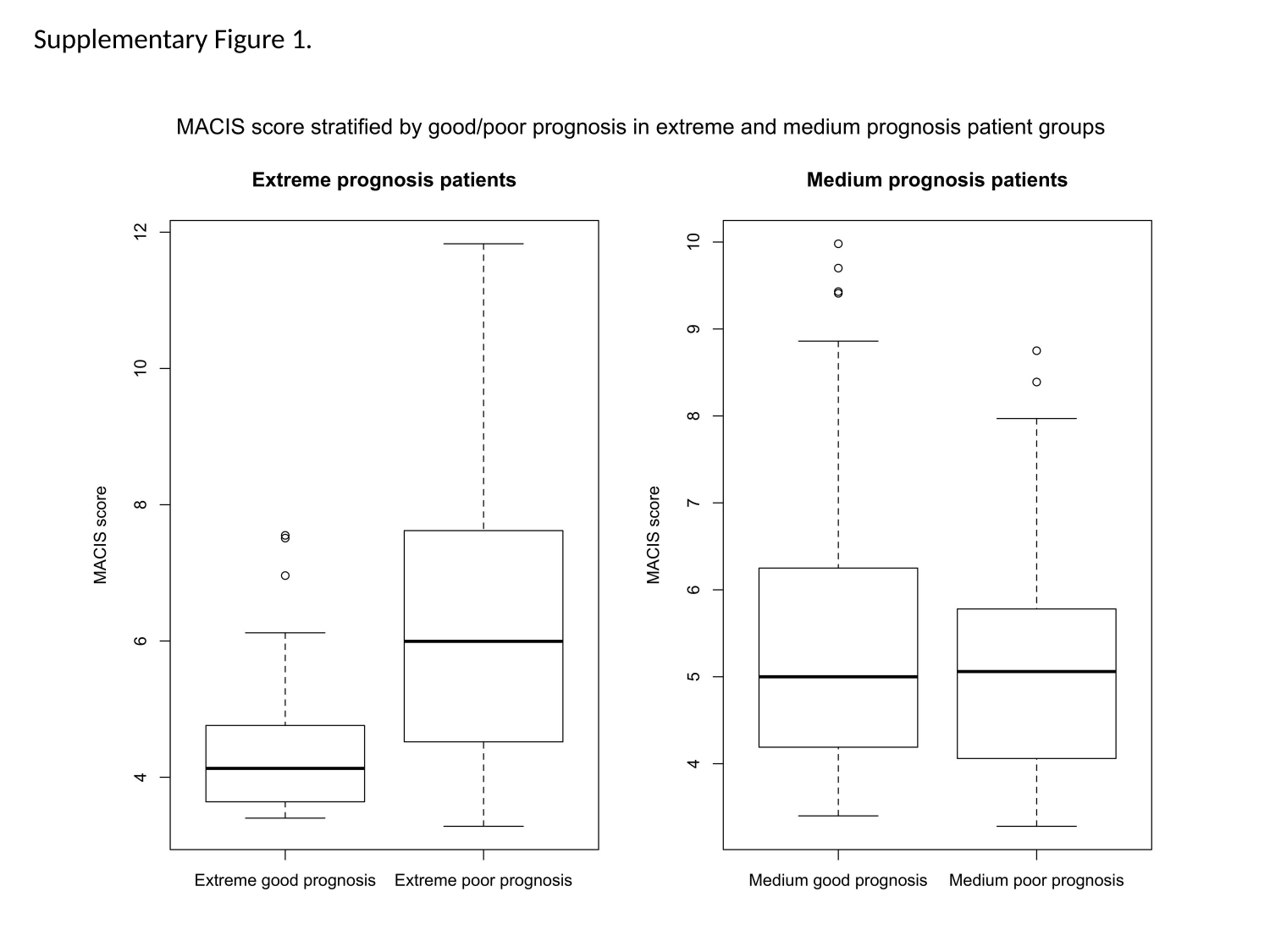

Supplementary Figure 1.

## Slide 2
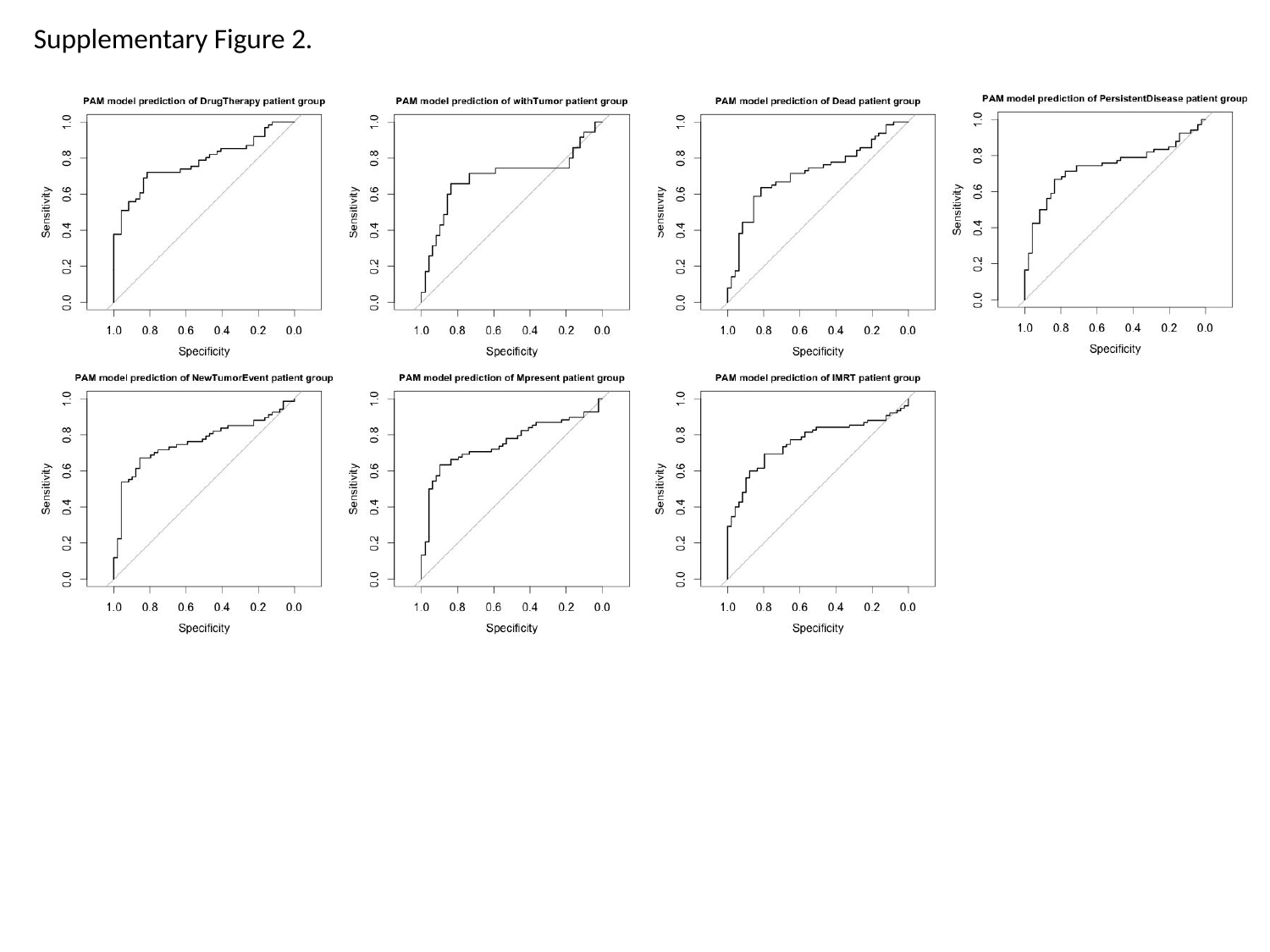

Supplementary Figure 2.

## Slide 3
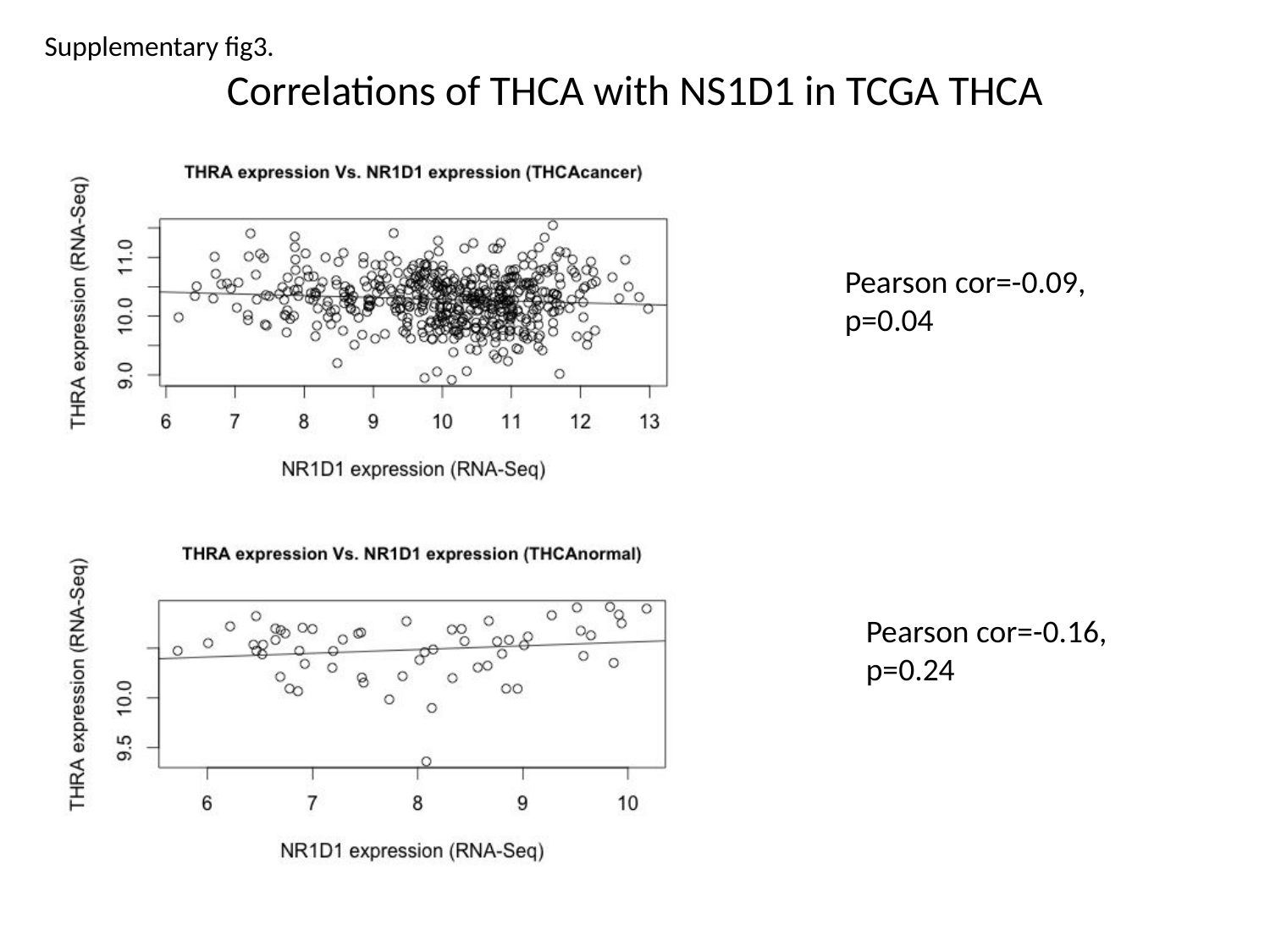

Supplementary fig3.
# Correlations of THCA with NS1D1 in TCGA THCA
Pearson cor=-0.09, p=0.04
Pearson cor=-0.16, p=0.24
